# Supplementary figures and images for: Associations between sexual habits, menstrual hygiene practices, demographics and the vaginal microbiome as revealed by Bayesian network analysis
Source: PLoS One. 2018 Jan 24;13(1):e0191625. doi: 10.1371/journal.pone.0191625 (PMC5783405; doi:10.1371/journal.pone.0191625)

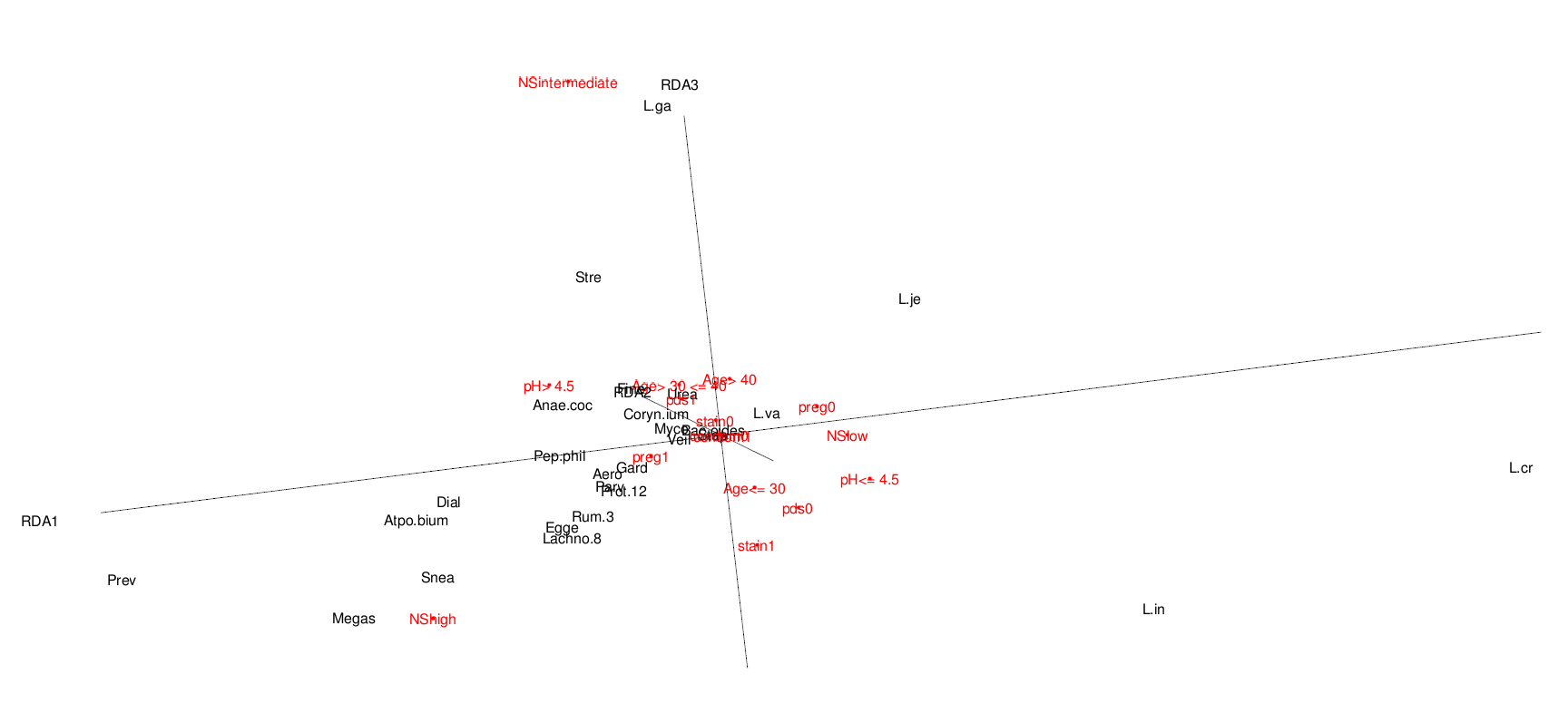

Supplement: S1 Fig — (TIFF) [file pone.0191625.s001.tiff]

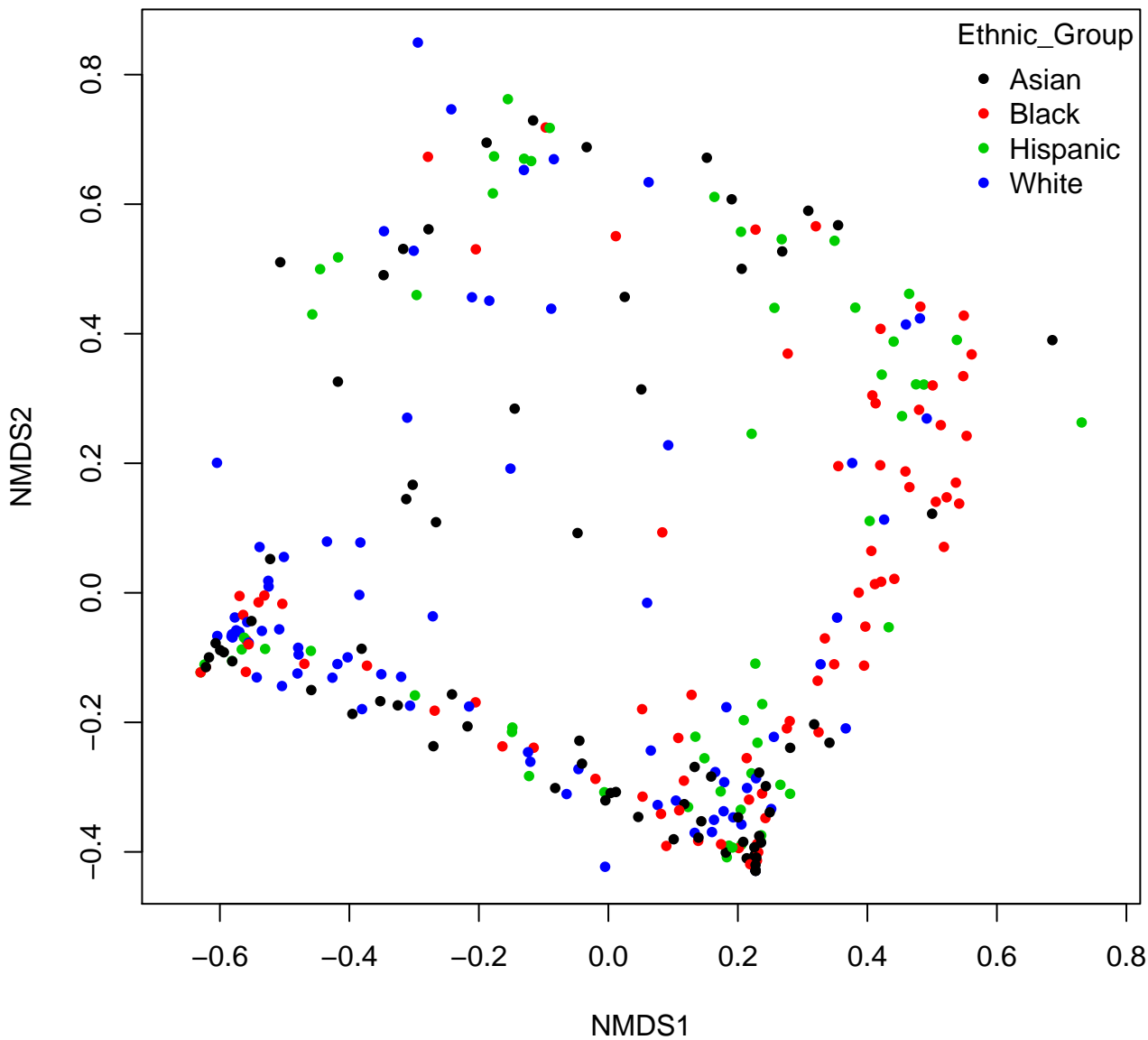

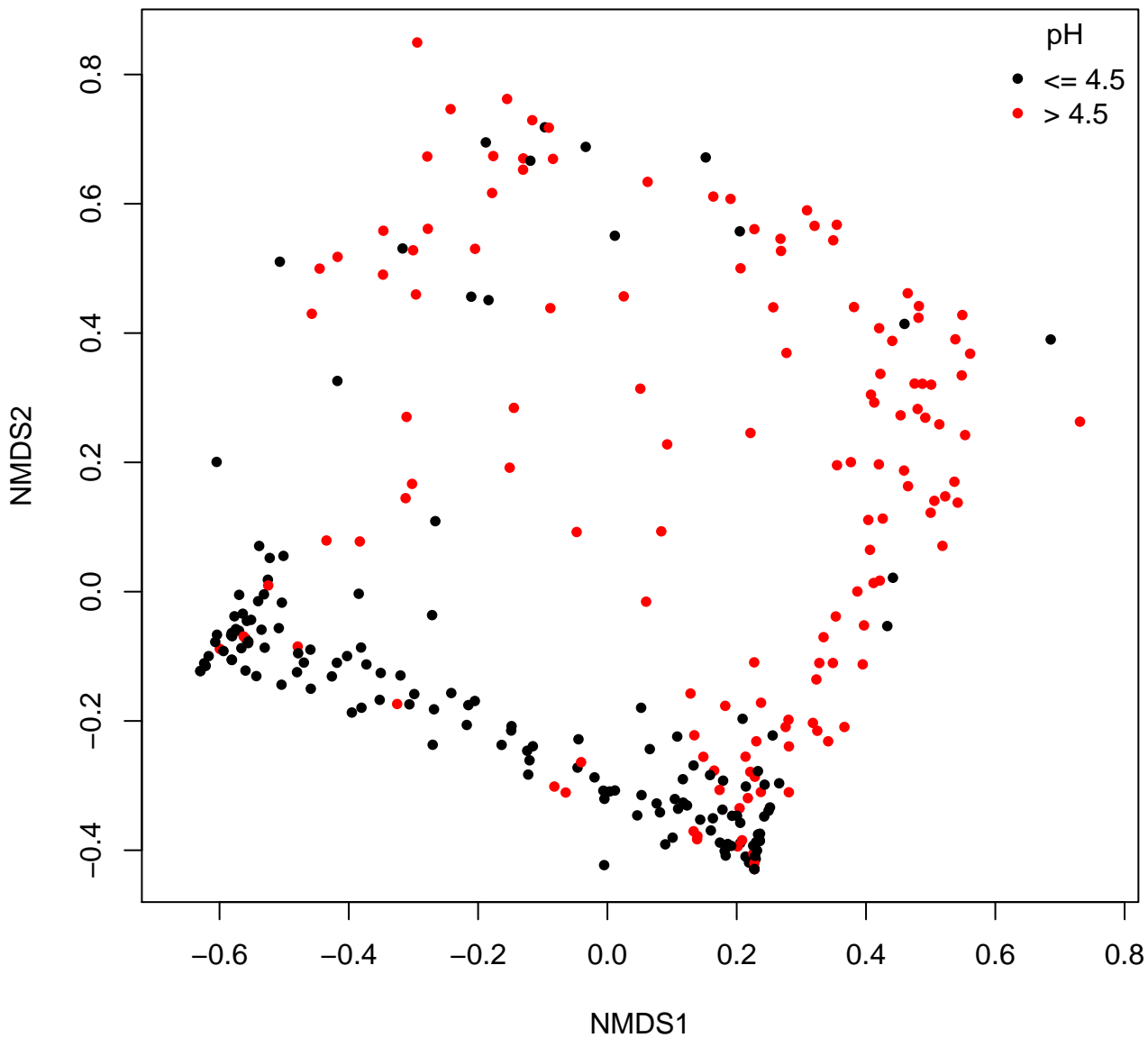

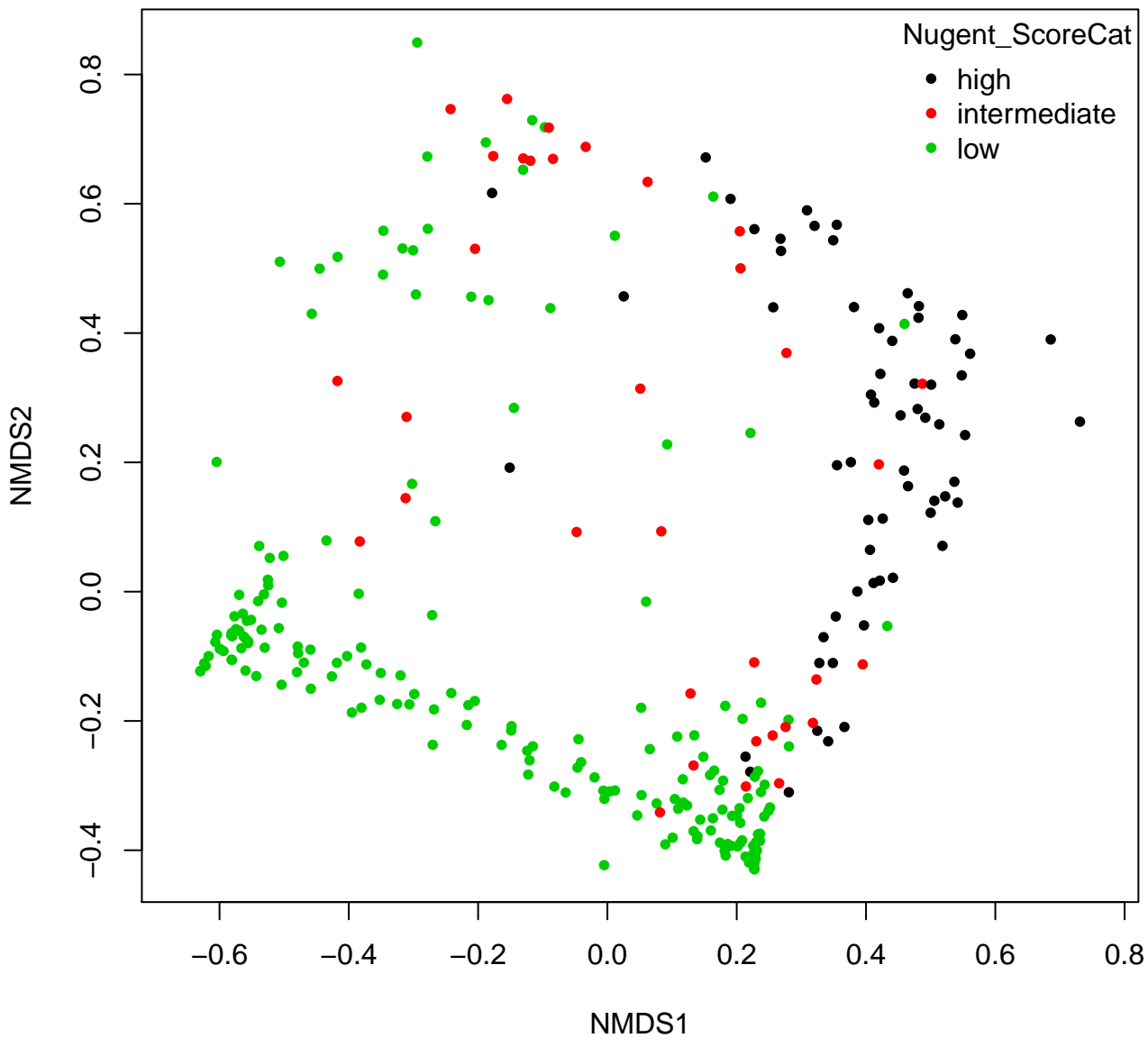

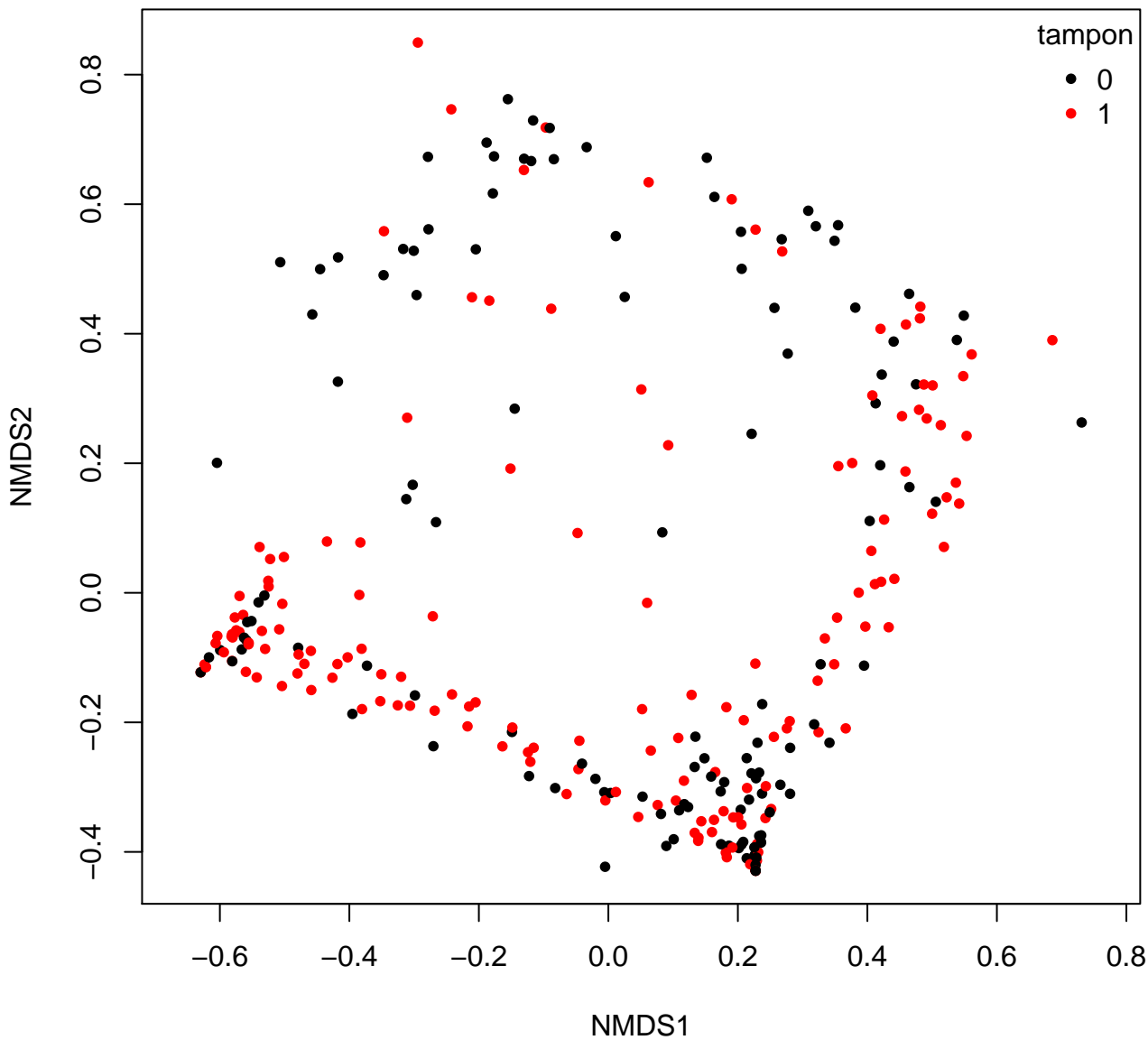

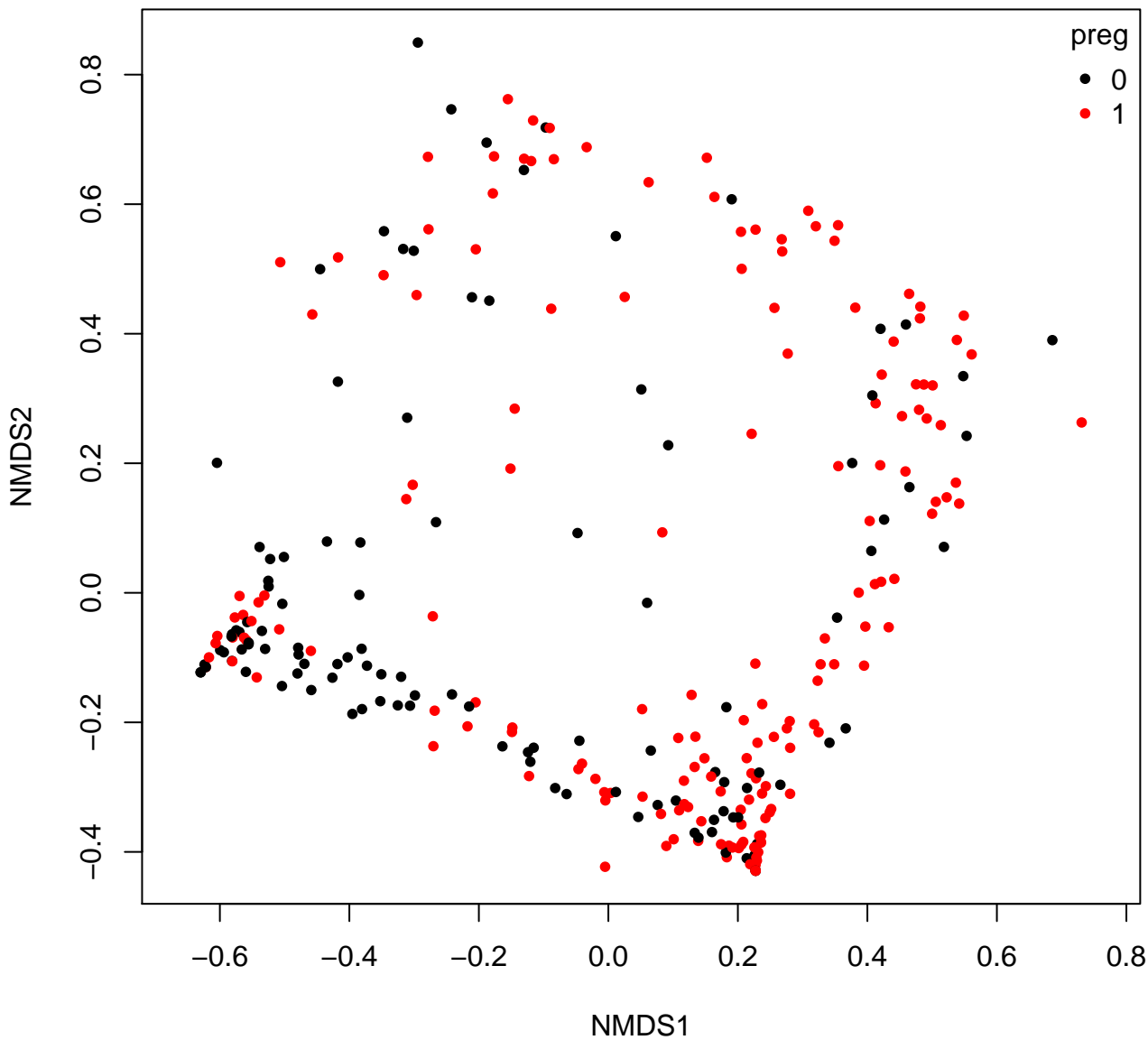

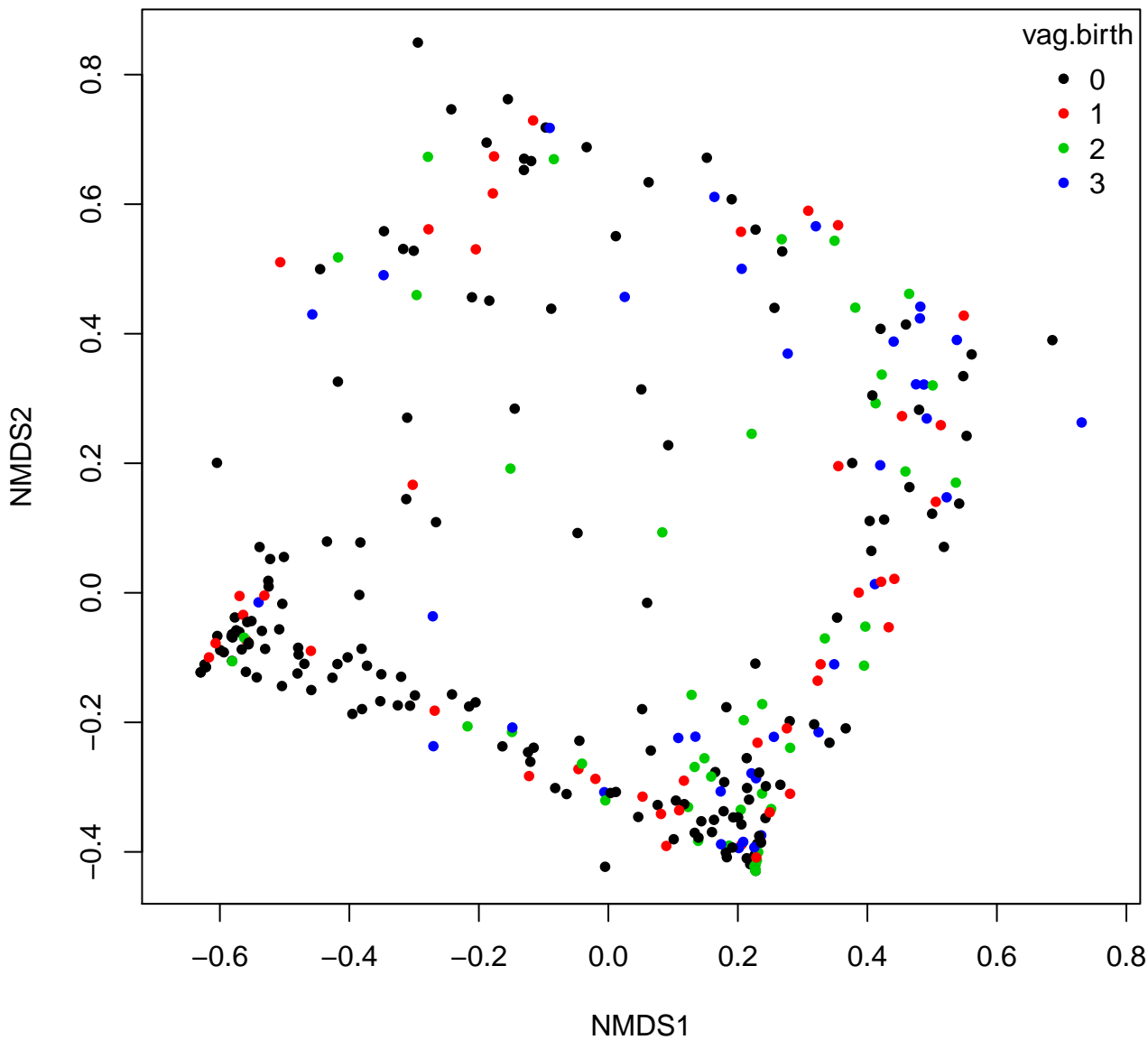

Supplement: S6 File — (PDF) [file pone.0191625.s007.pdf]
