# Supplementary material for: Associations between sexual habits, menstrual hygiene practices, demographics and the vaginal microbiome as revealed by Bayesian network analysis
Source: PLoS One. 2018 Jan 24;13(1):e0191625. doi: 10.1371/journal.pone.0191625 (PMC5783405; doi:10.1371/journal.pone.0191625)
Supplement: S7 File — (PDF) [file pone.0191625.s008.pdf]

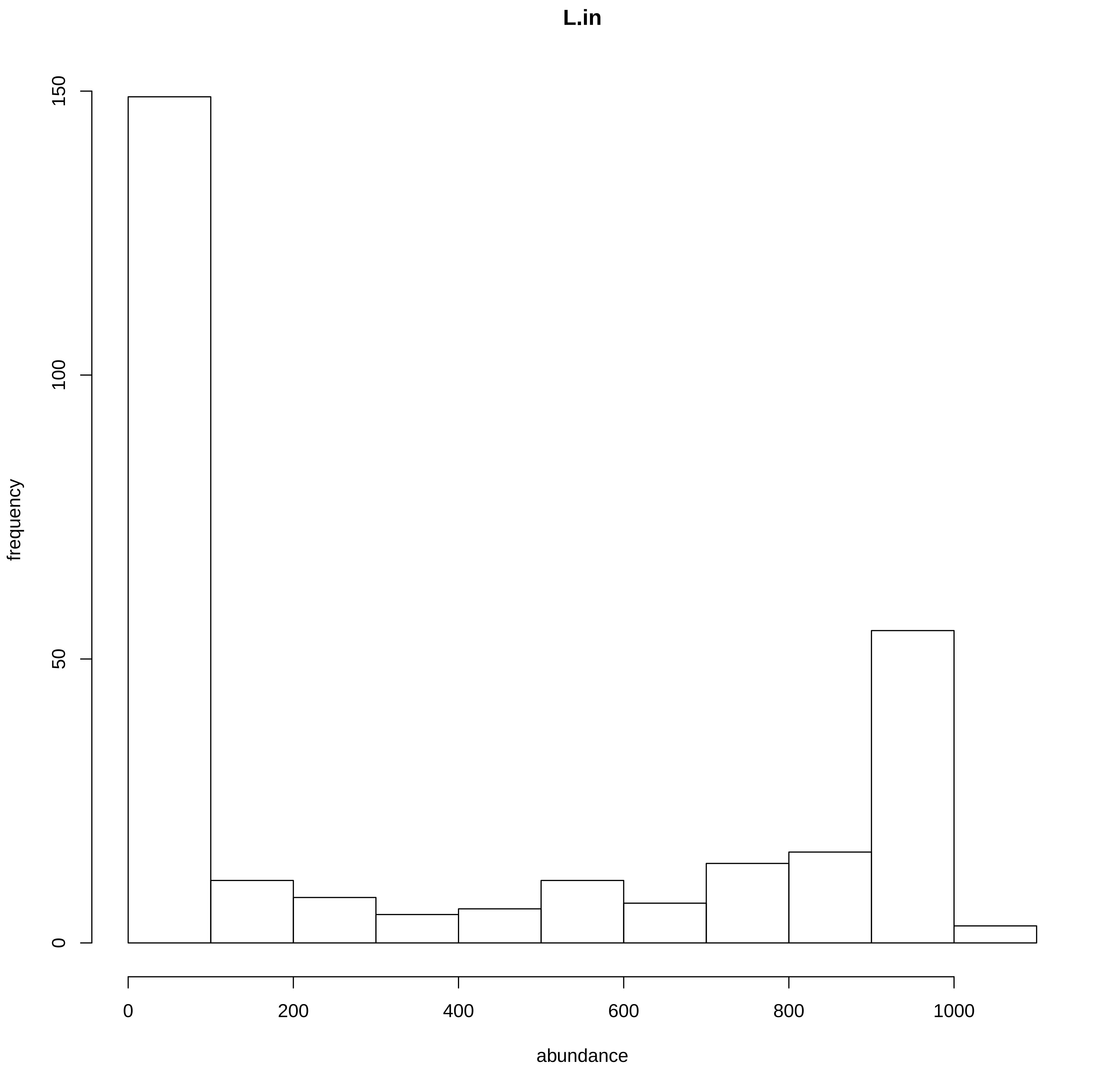
L.in
frequency
0
50
100
150
0
200
400
600
800
1000
abundance

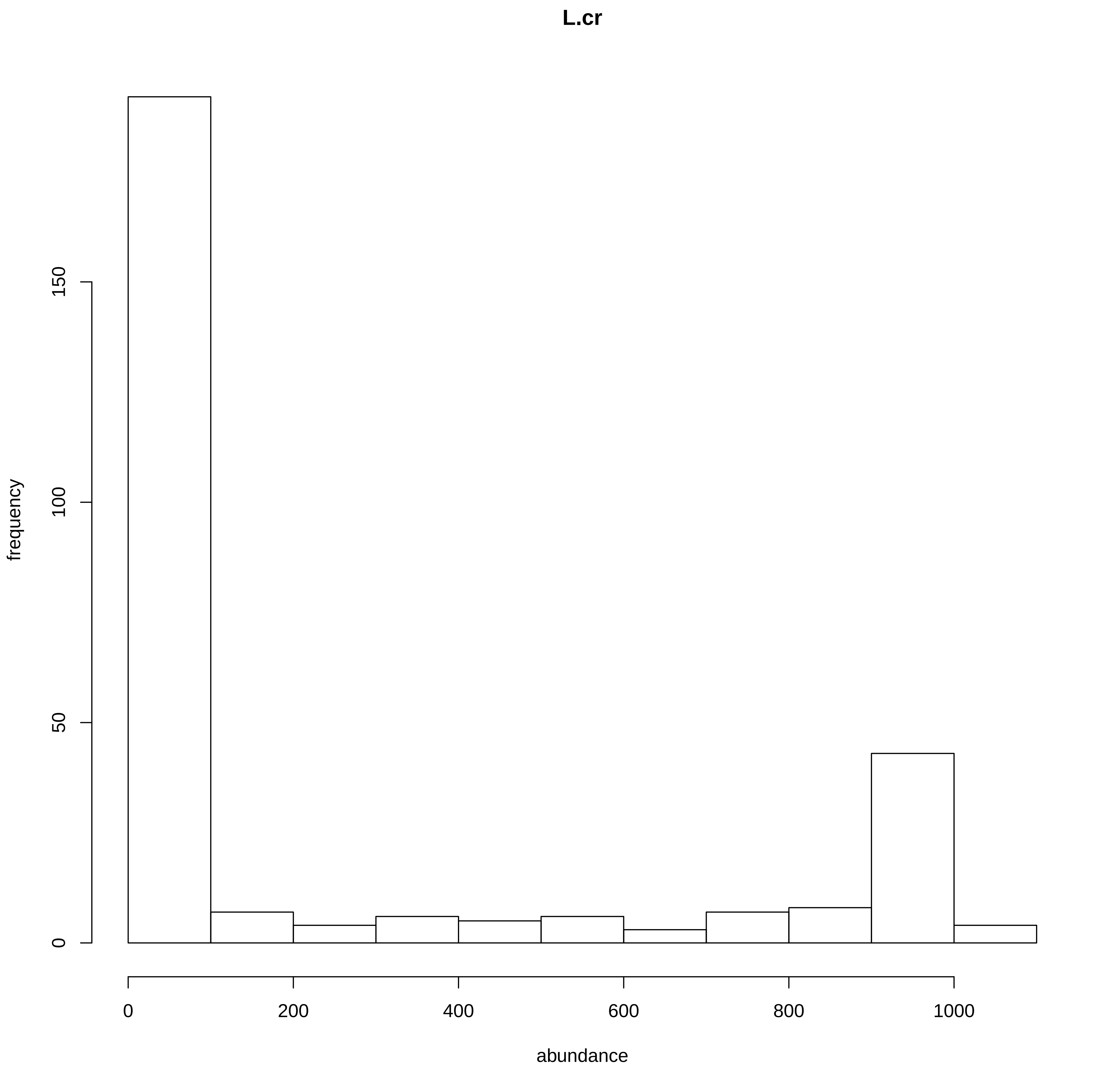

L.cr
frequency
0
50
100
150
0
200
400
600
800
1000
abundance

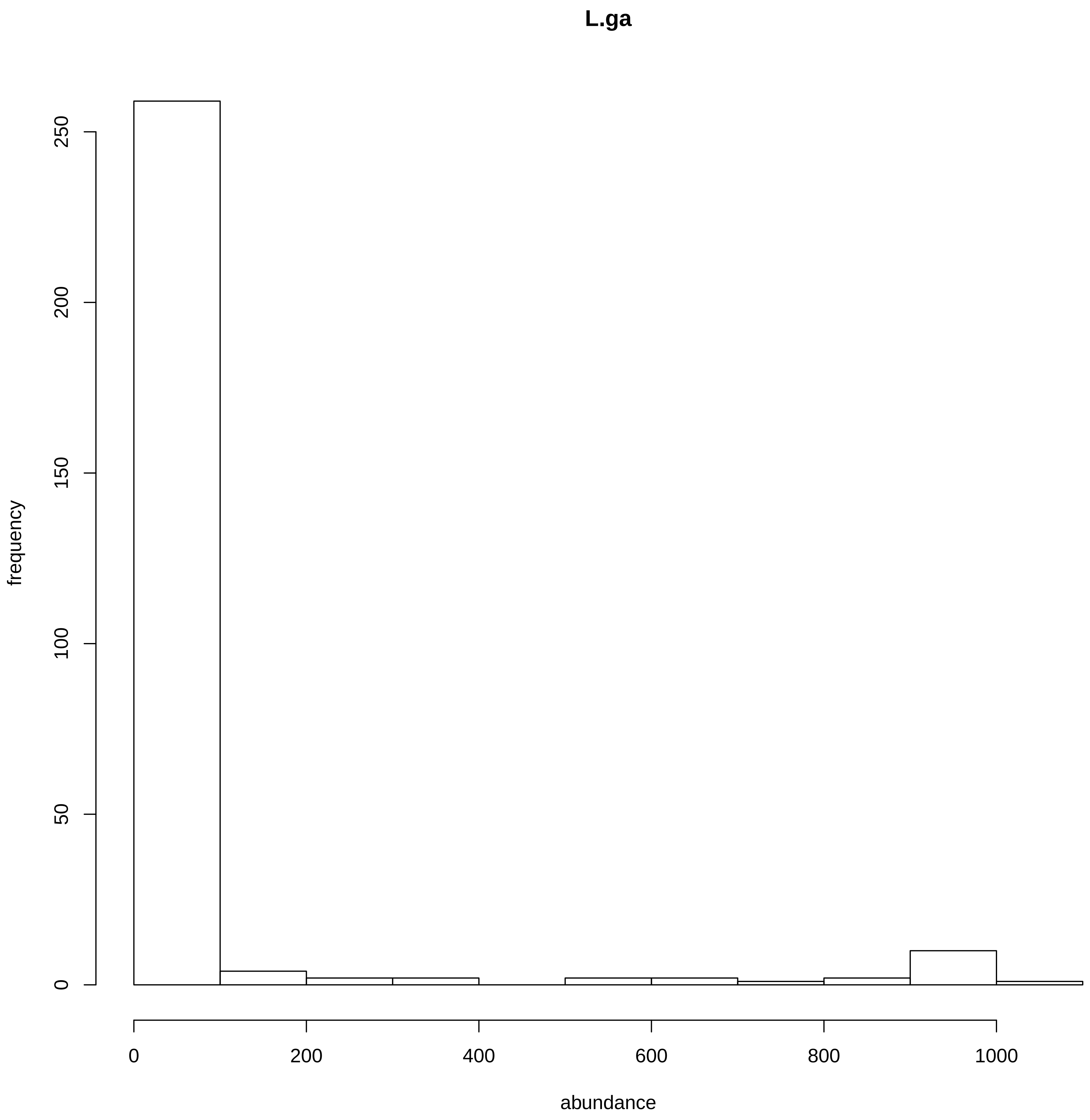
L.ga
frequency
0
50
100
150
200
250
0
200
400
600
800
1000
abundance

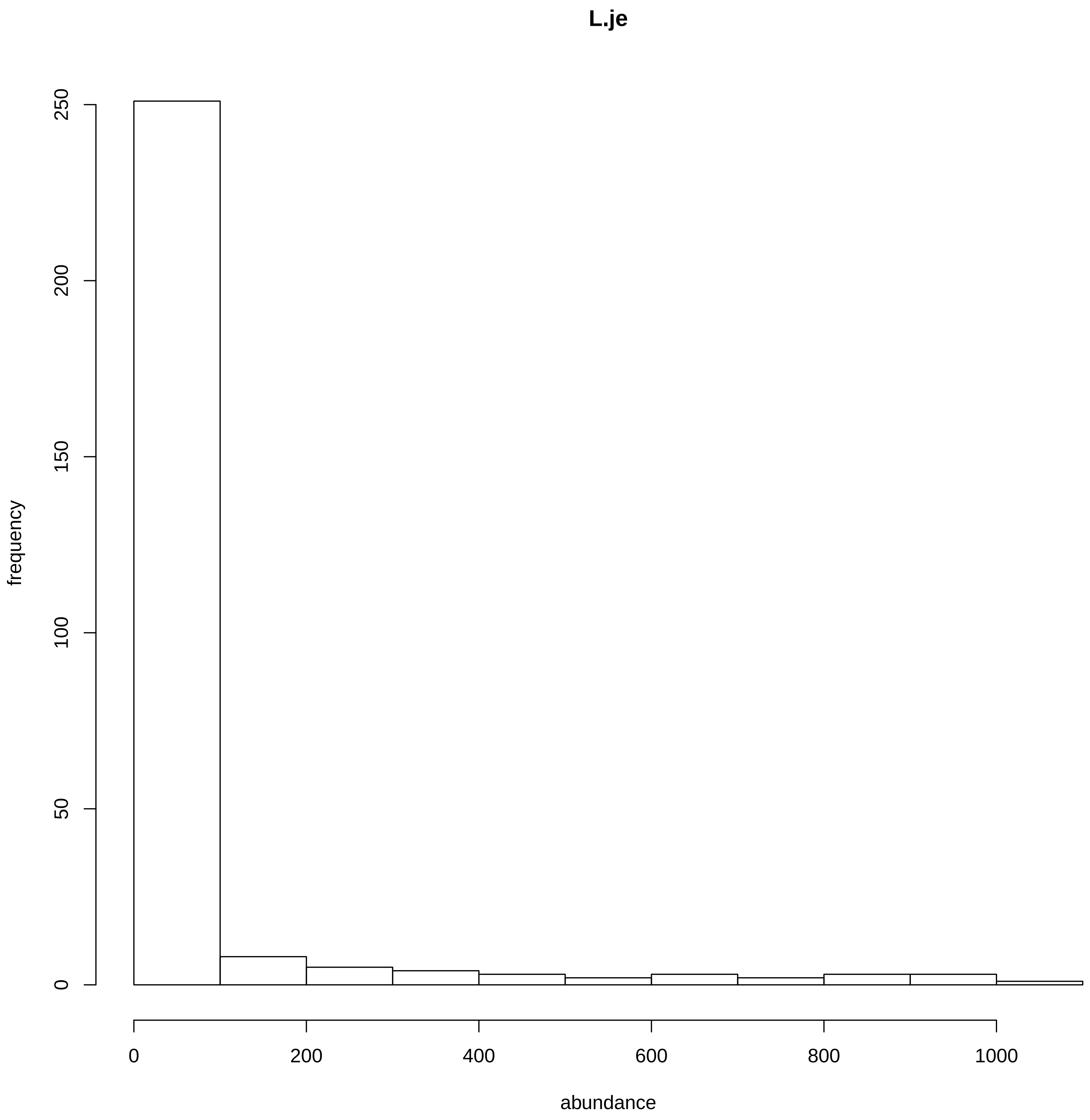

L.je
frequency
0
50
100
150
200
250
0
200
400
600
800
1000
abundance

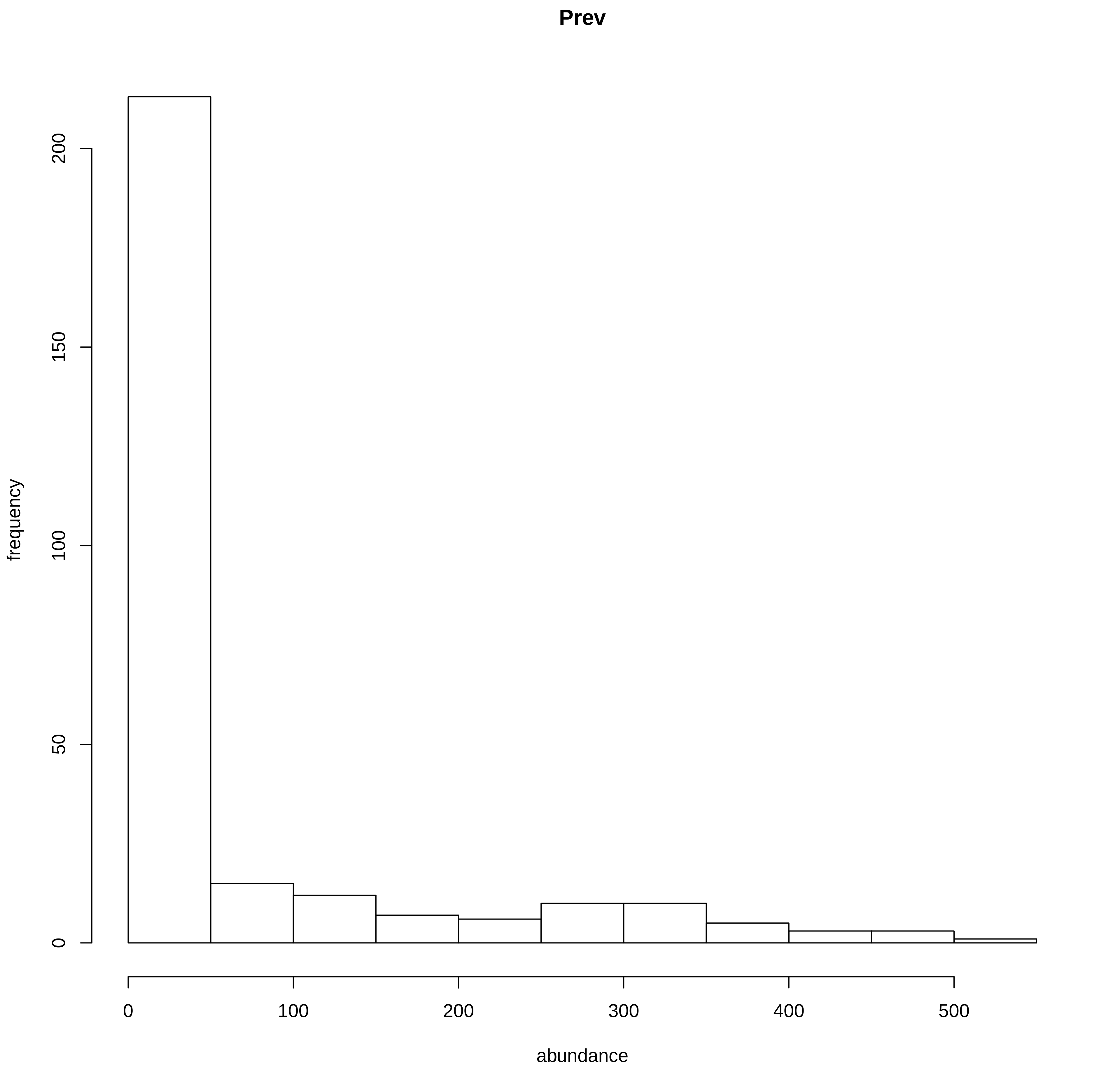
Prev
frequency
0
50
100
150
200
0
100
200
300
400
500
abundance

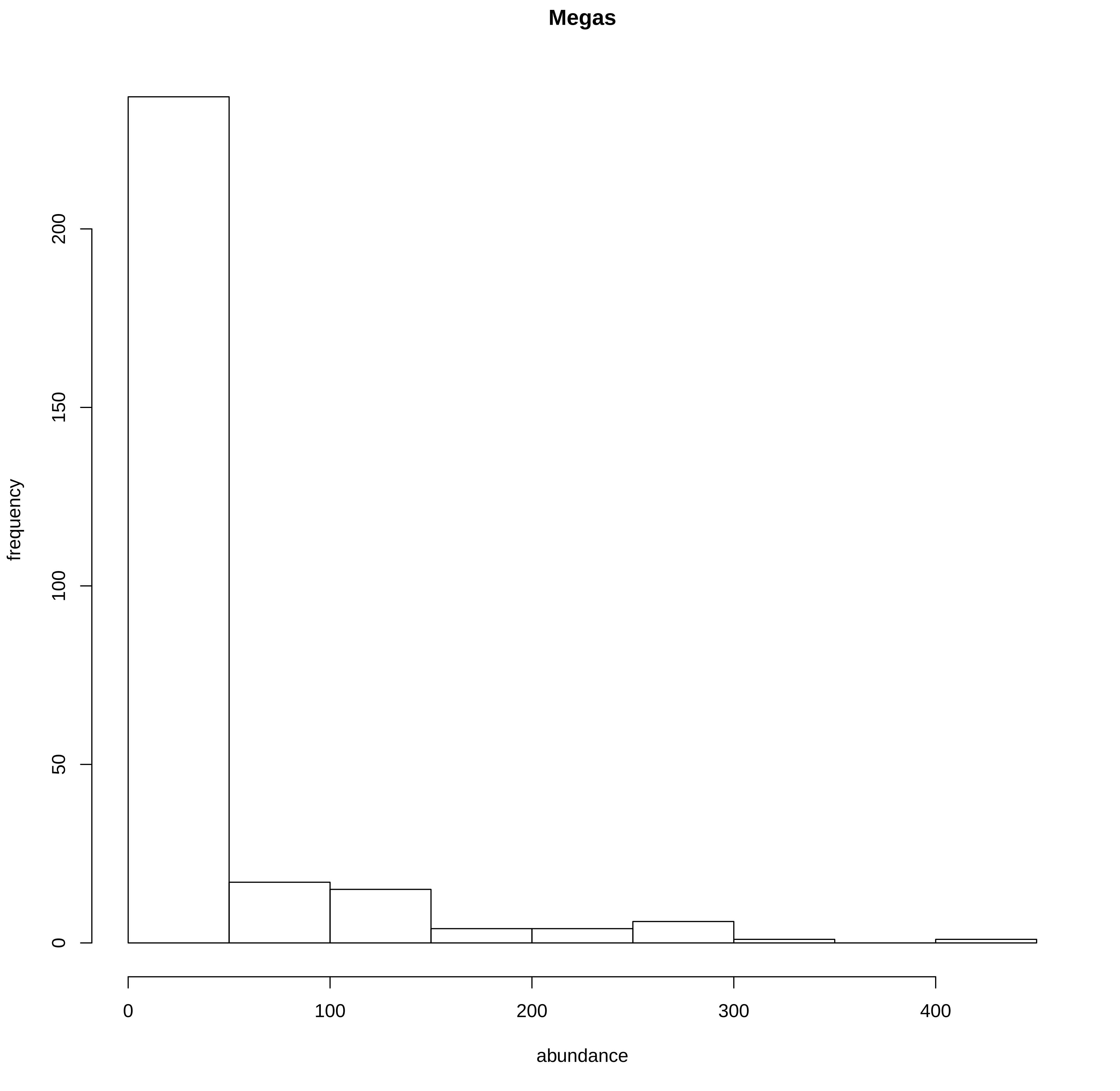

Megas
frequency
abundance
0
50
100
150
200
0
100
200
300
400

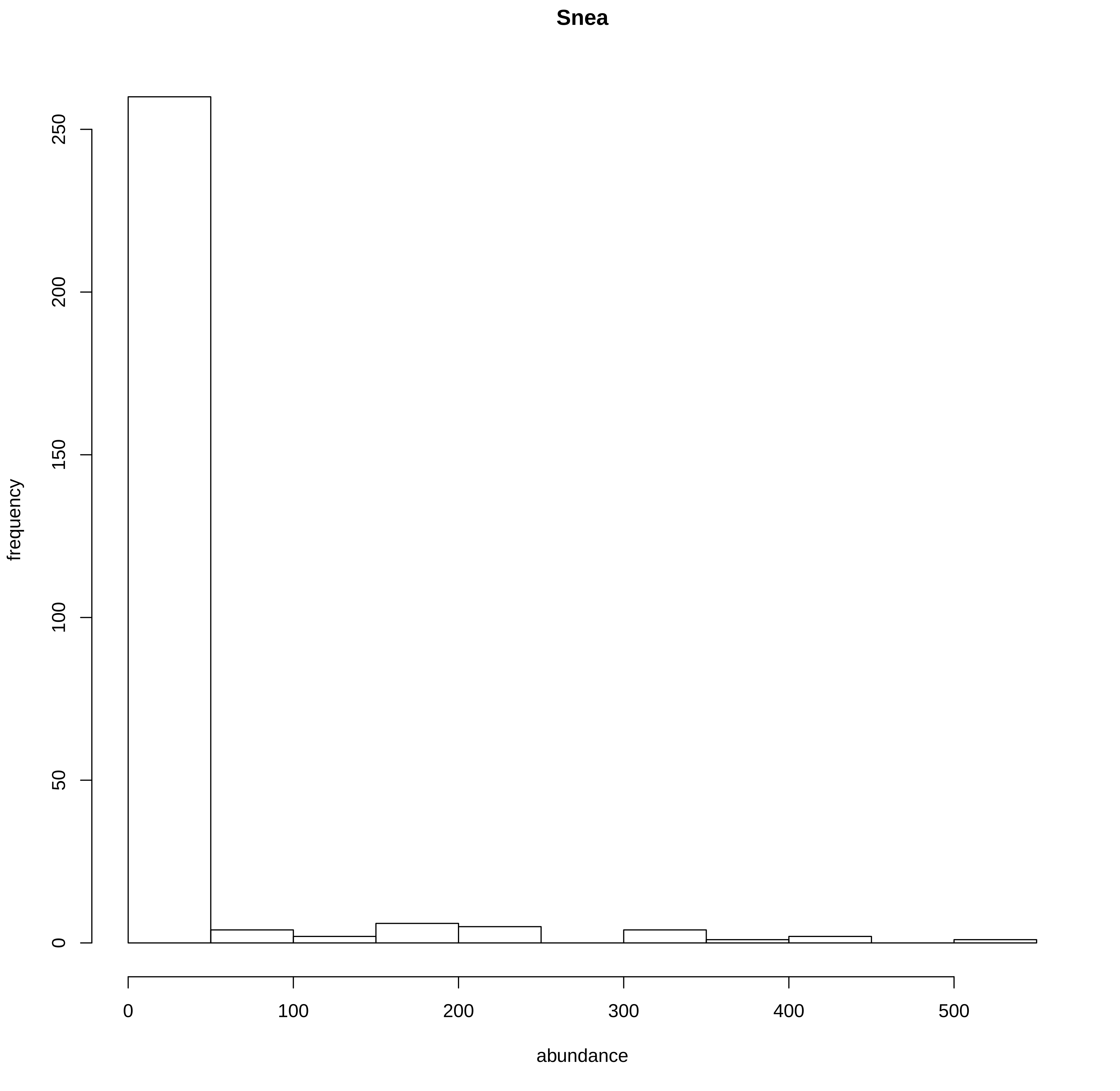
Snea
frequency
0
50
100
150
200
250
0
100
200
300
400
500
abundance

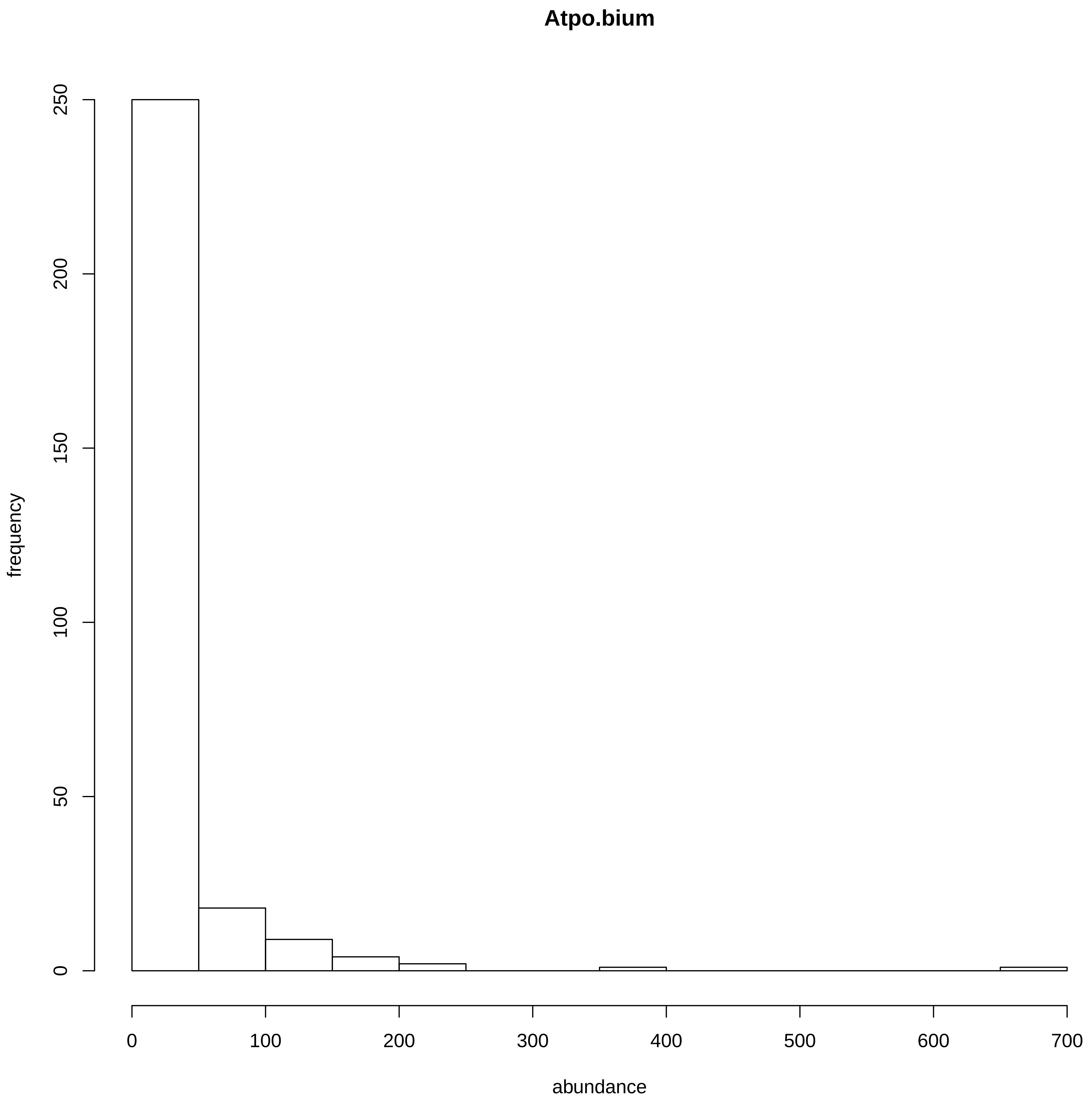

Atpo.bium
frequency
0
50
100
150
200
250
0
100
200
300
400
500
600
700
abundance

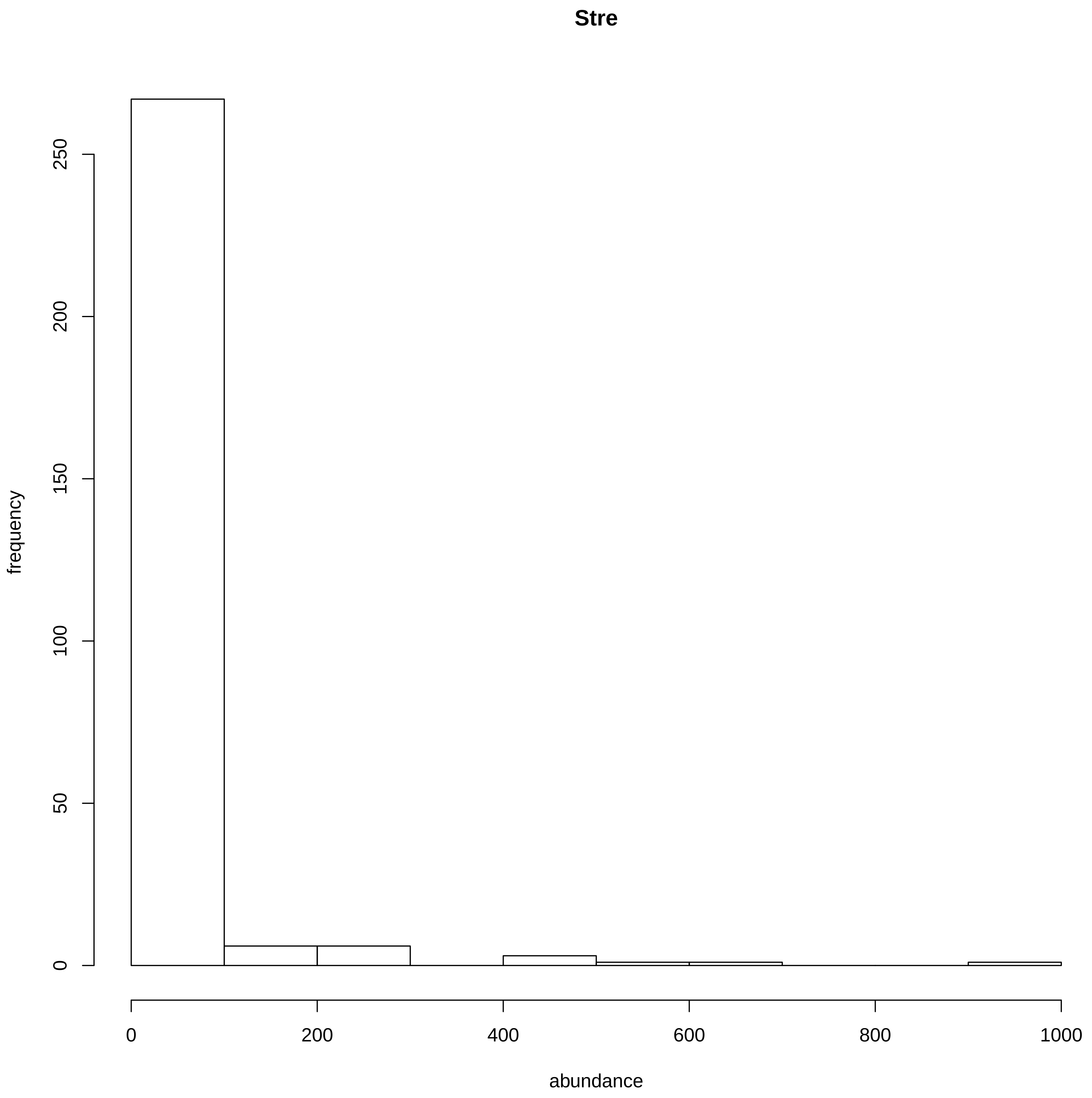

Stre
frequency
0
50
100
150
200
250
0
200
400
600
800
1000
abundance

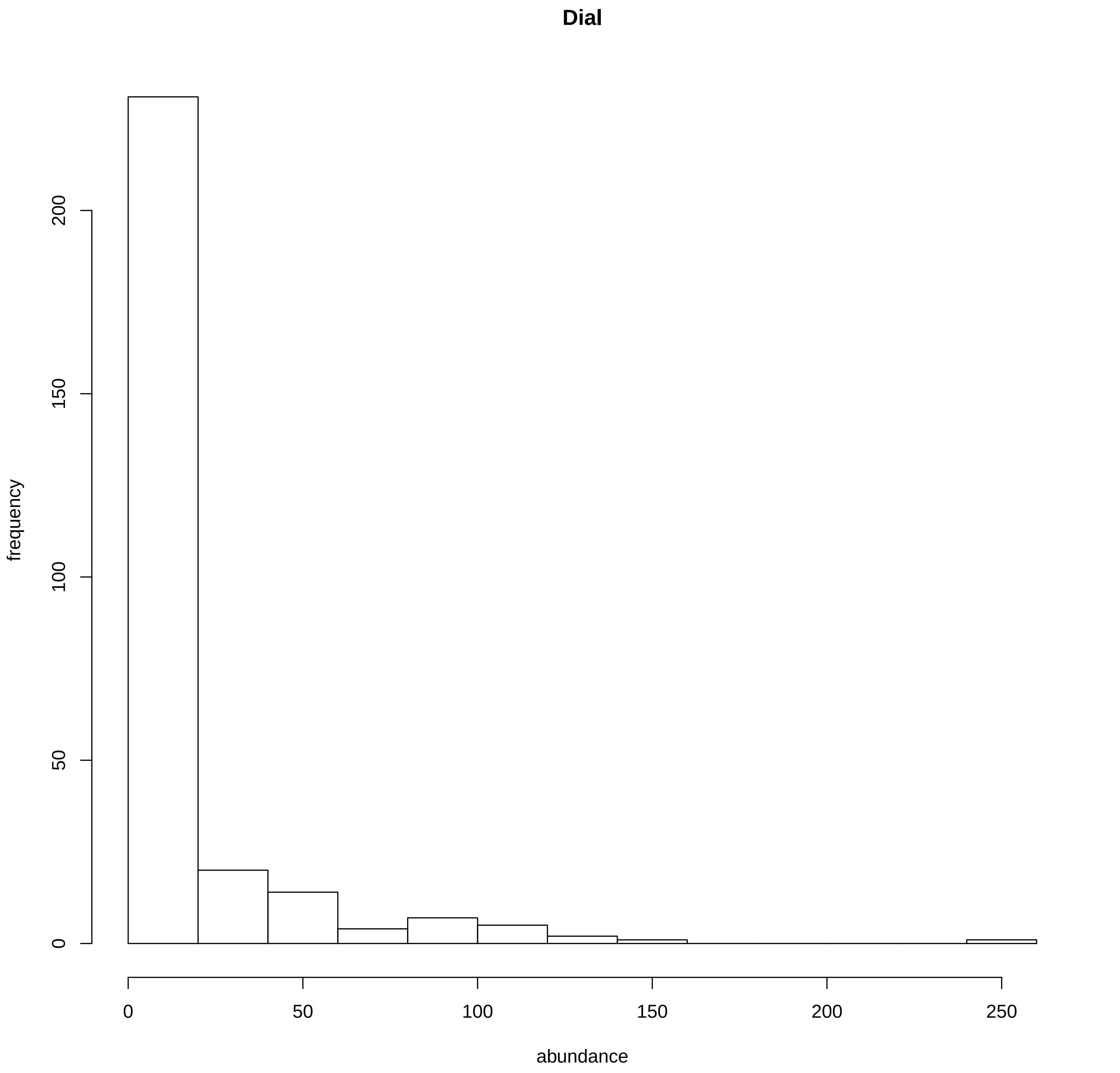

Dial
frequency
0
50
100
150
200
0
50
100
150
200
250
abundance

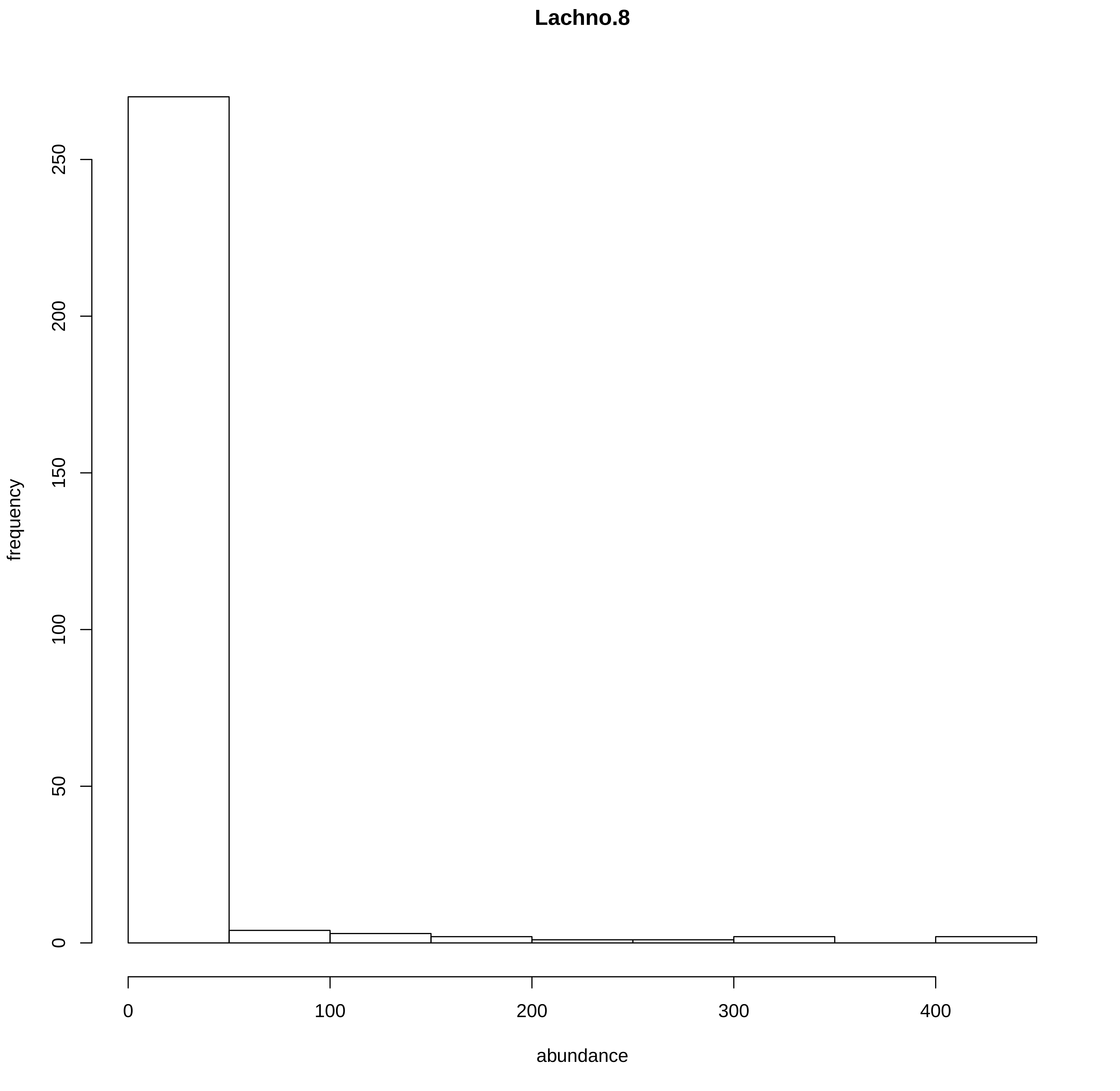
Lachno.8
frequency
0
50
100
150
200
250
0
100
200
300
400
abundance

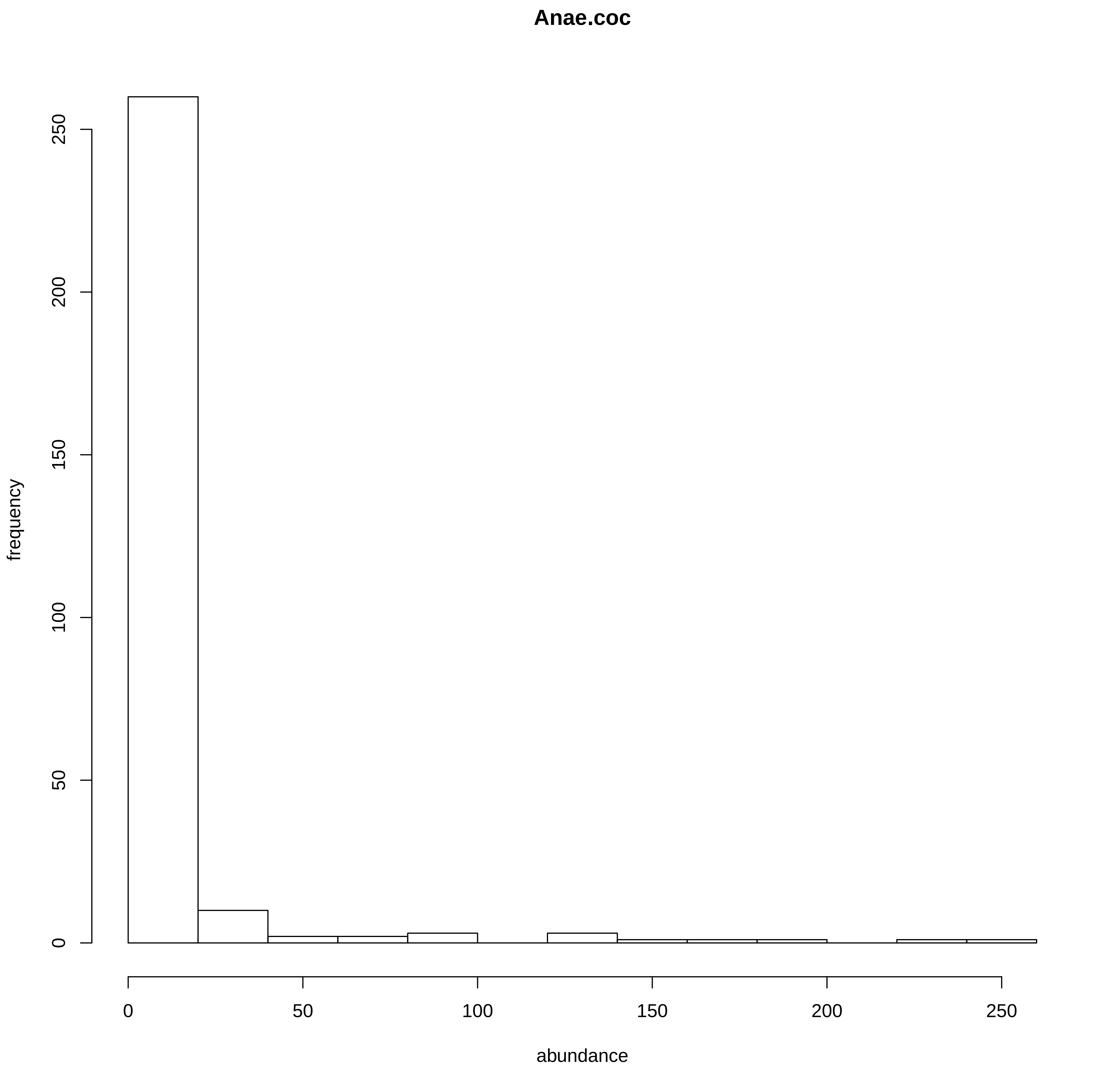

Anae.coc
frequency
0
50
100
150
200
250
0
50
100
150
200
250
abundance

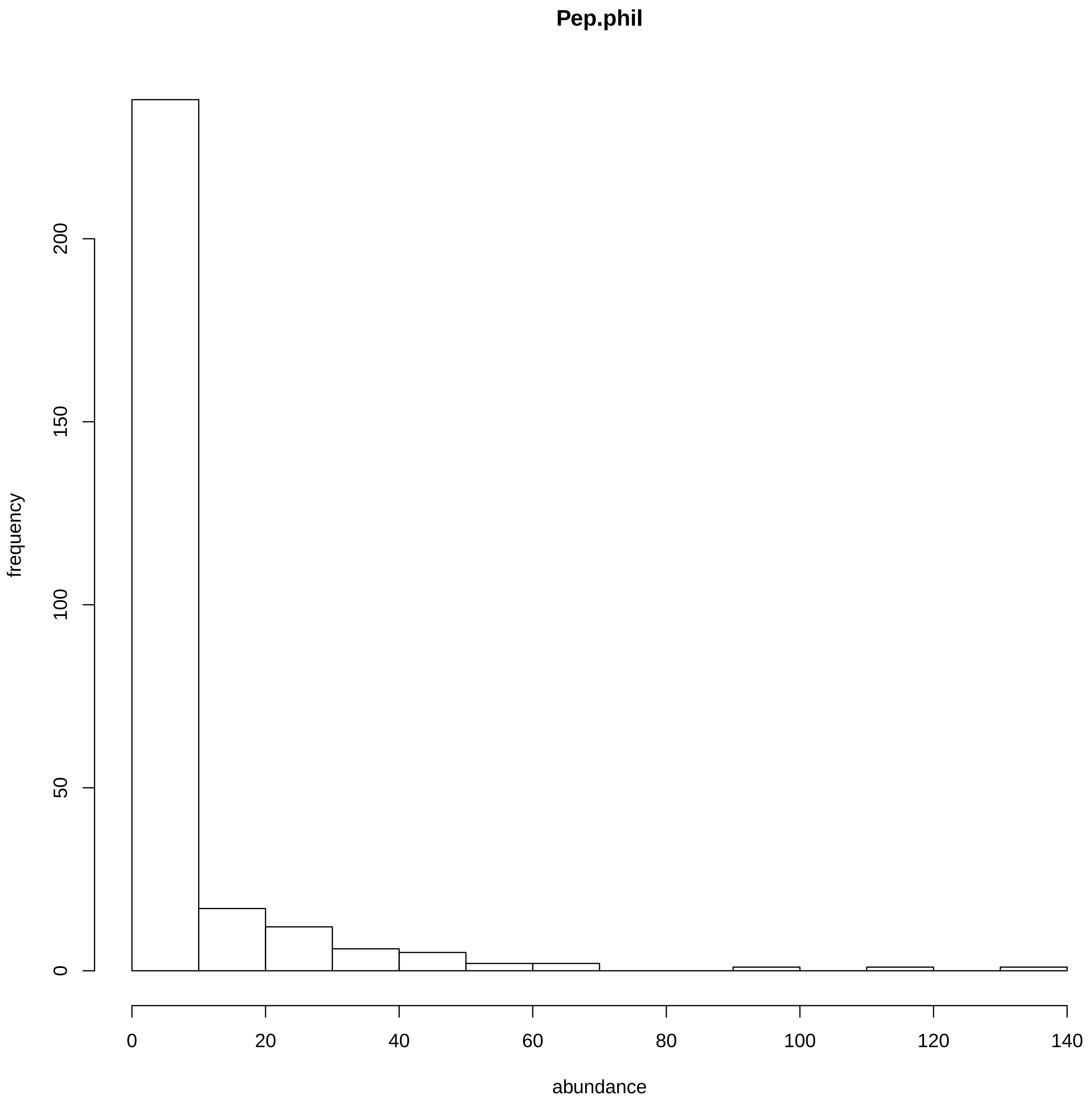
Pep.phil
frequency
0
50
100
150
200
0
20
40
60
80
100
120
140
abundance

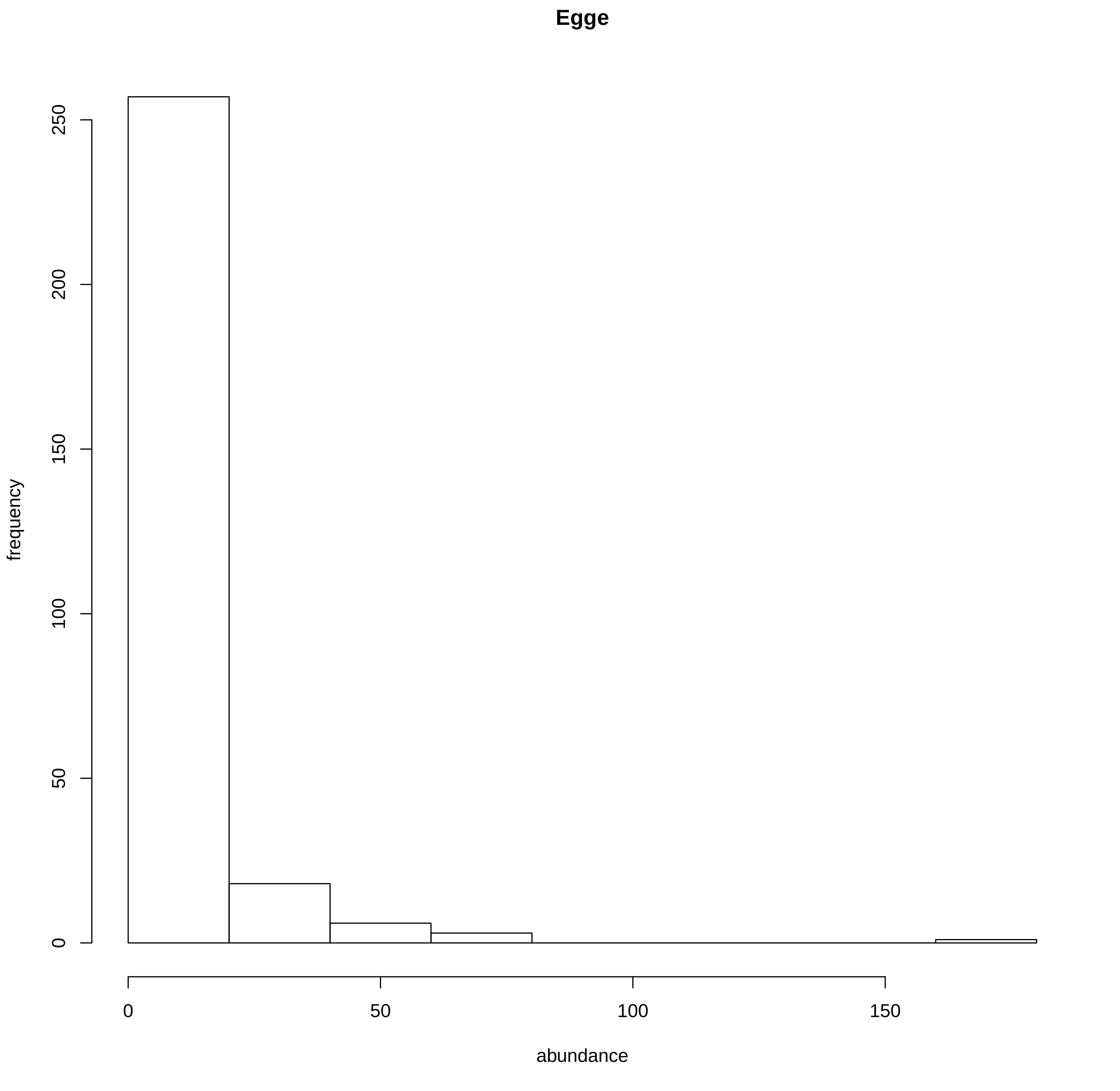
Egge
frequency
abundance
0
50
100
150
200
250
0
50
100
150

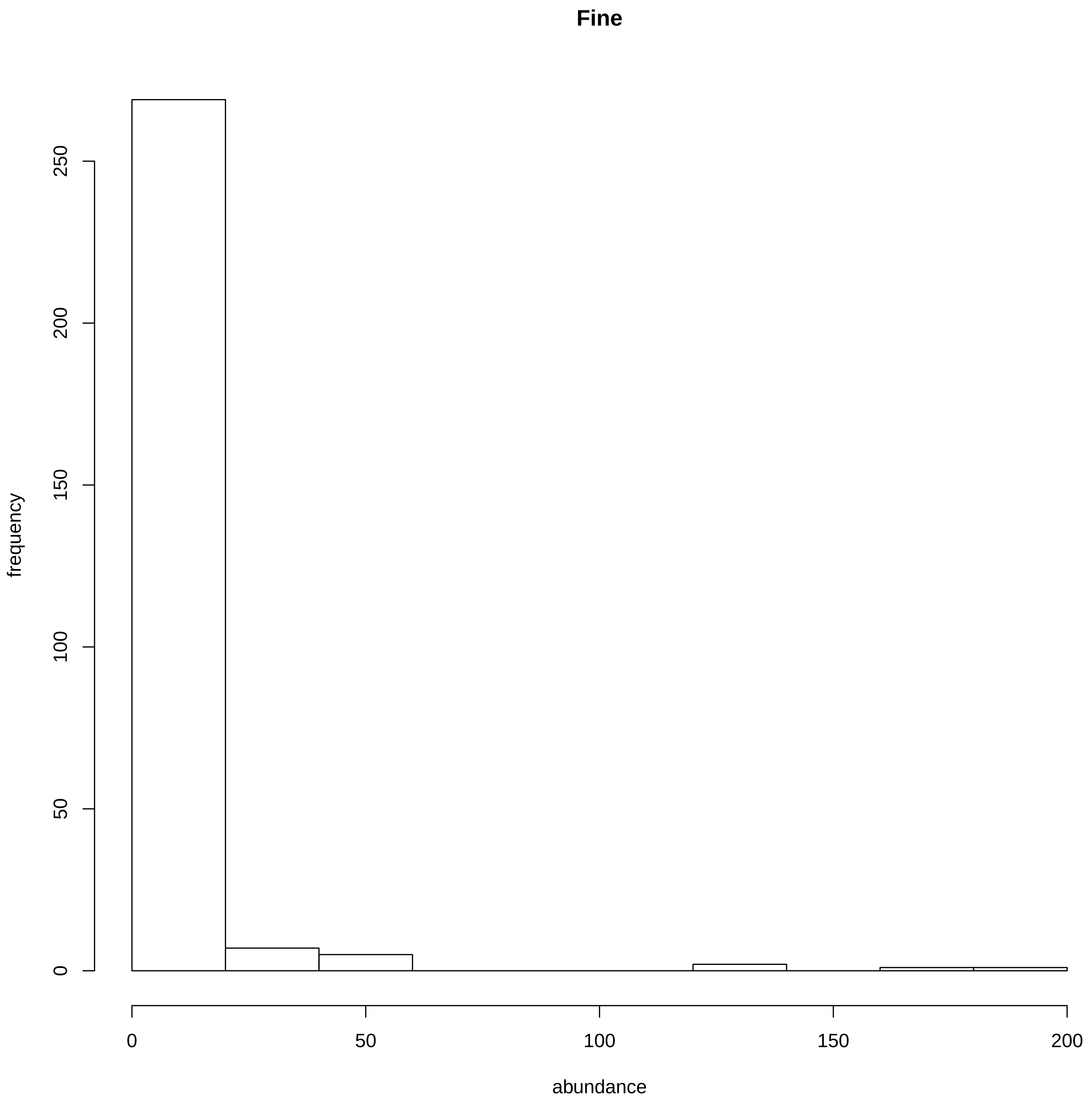

Fine
frequency
0
50
100
150
200
250
0
50
100
150
200
abundance

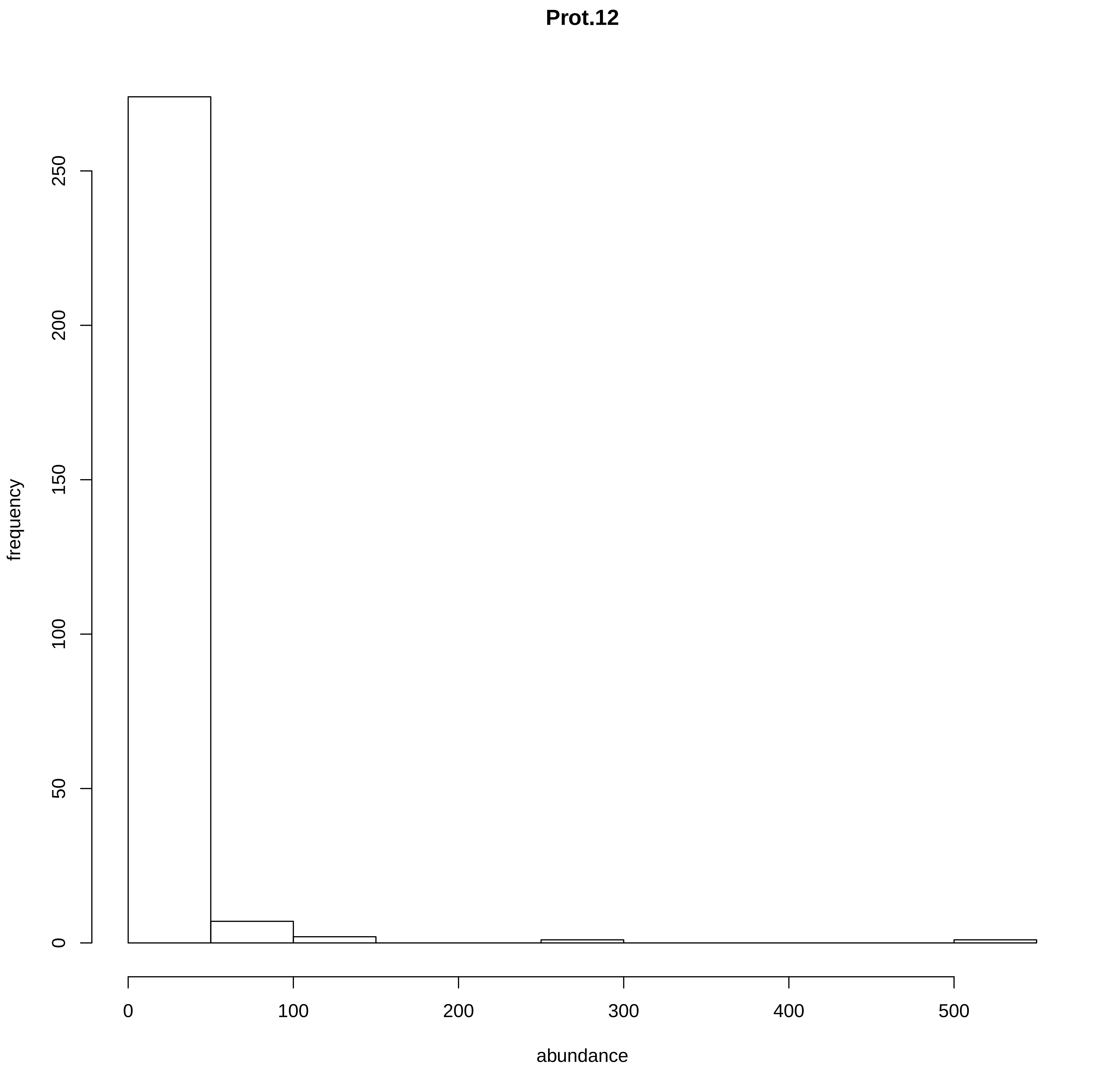
Prot.12
frequency
250
200
150
100
50
0
0
100
200
300
400
500
abundance

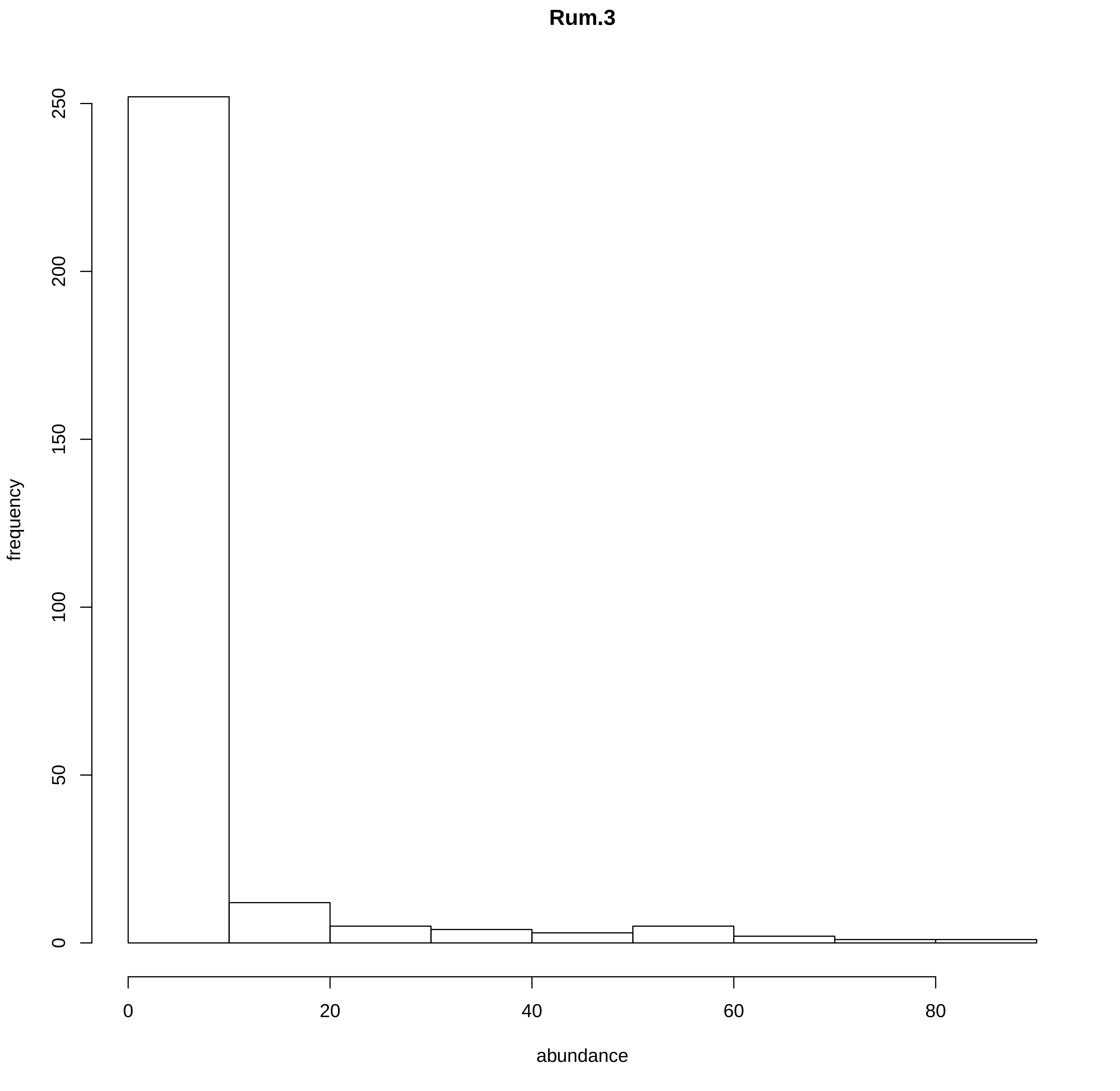

Rum.3
frequency
abundance
0
50
100
150
200
250
0
20
40
60
80

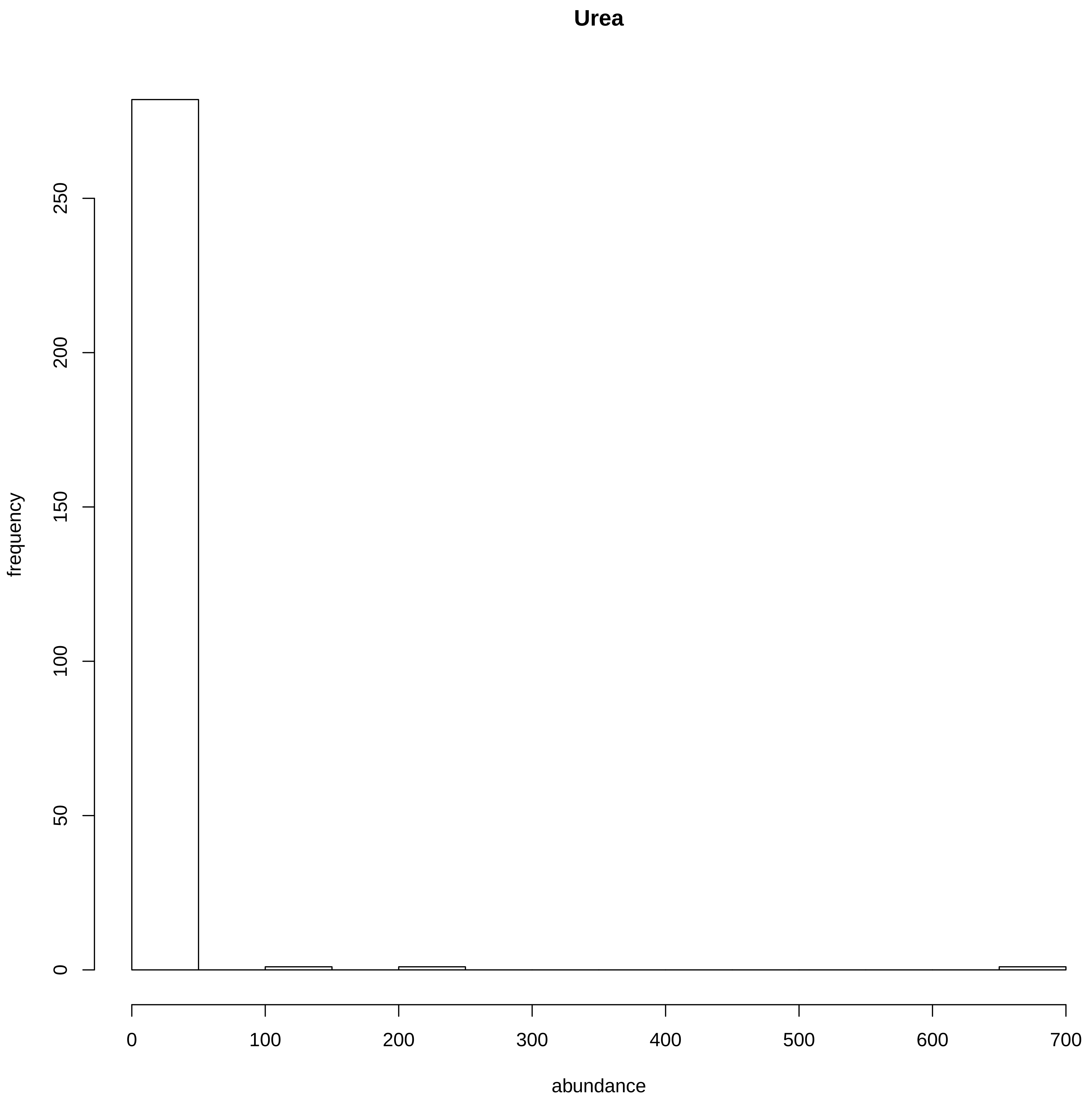
Urea
frequency
0
50
100
150
200
250
0
100
200
300
400
500
600
700
abundance

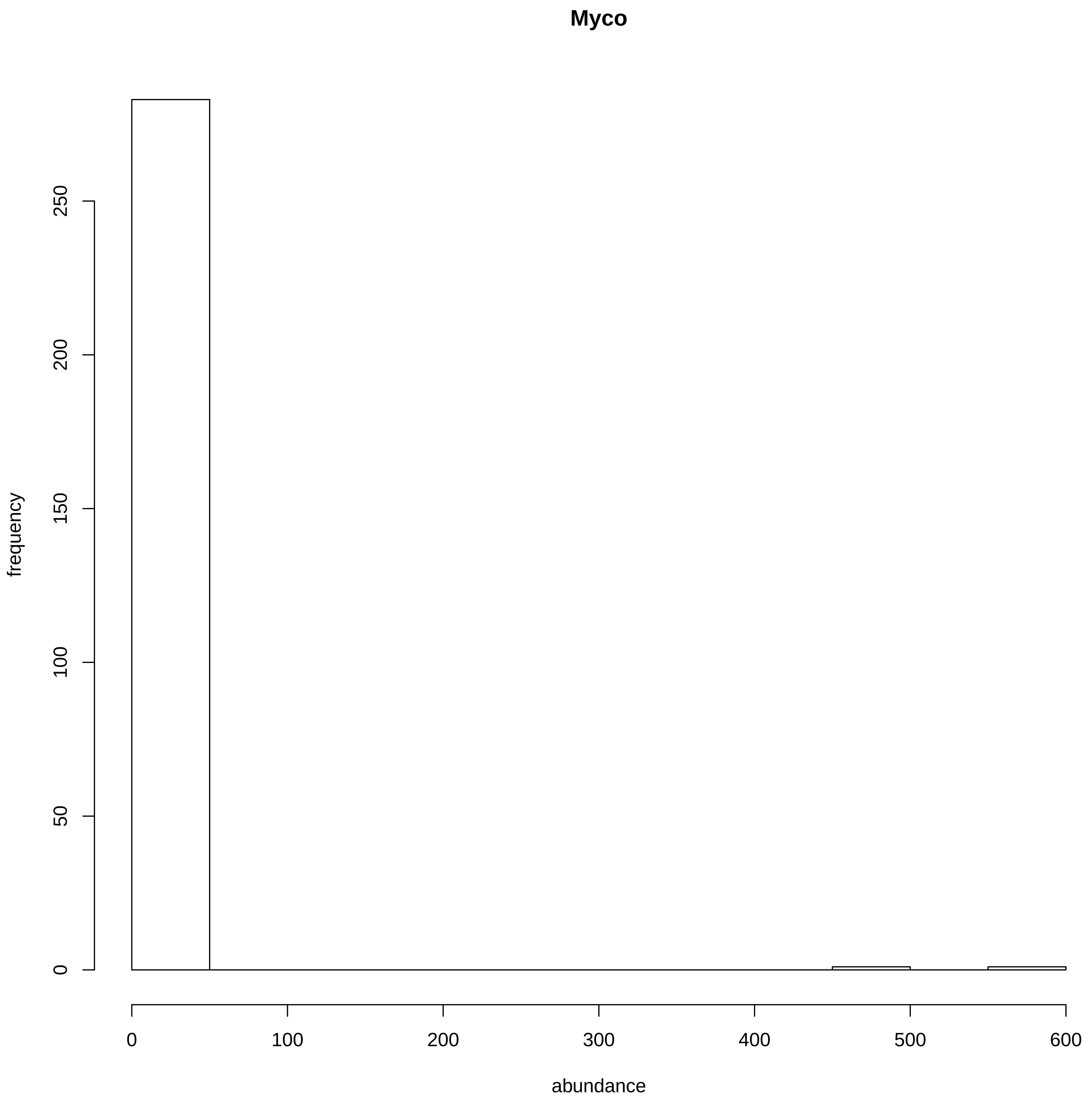
Myco
frequency
0
50
100
150
200
250
0
100
200
300
400
500
600
abundance

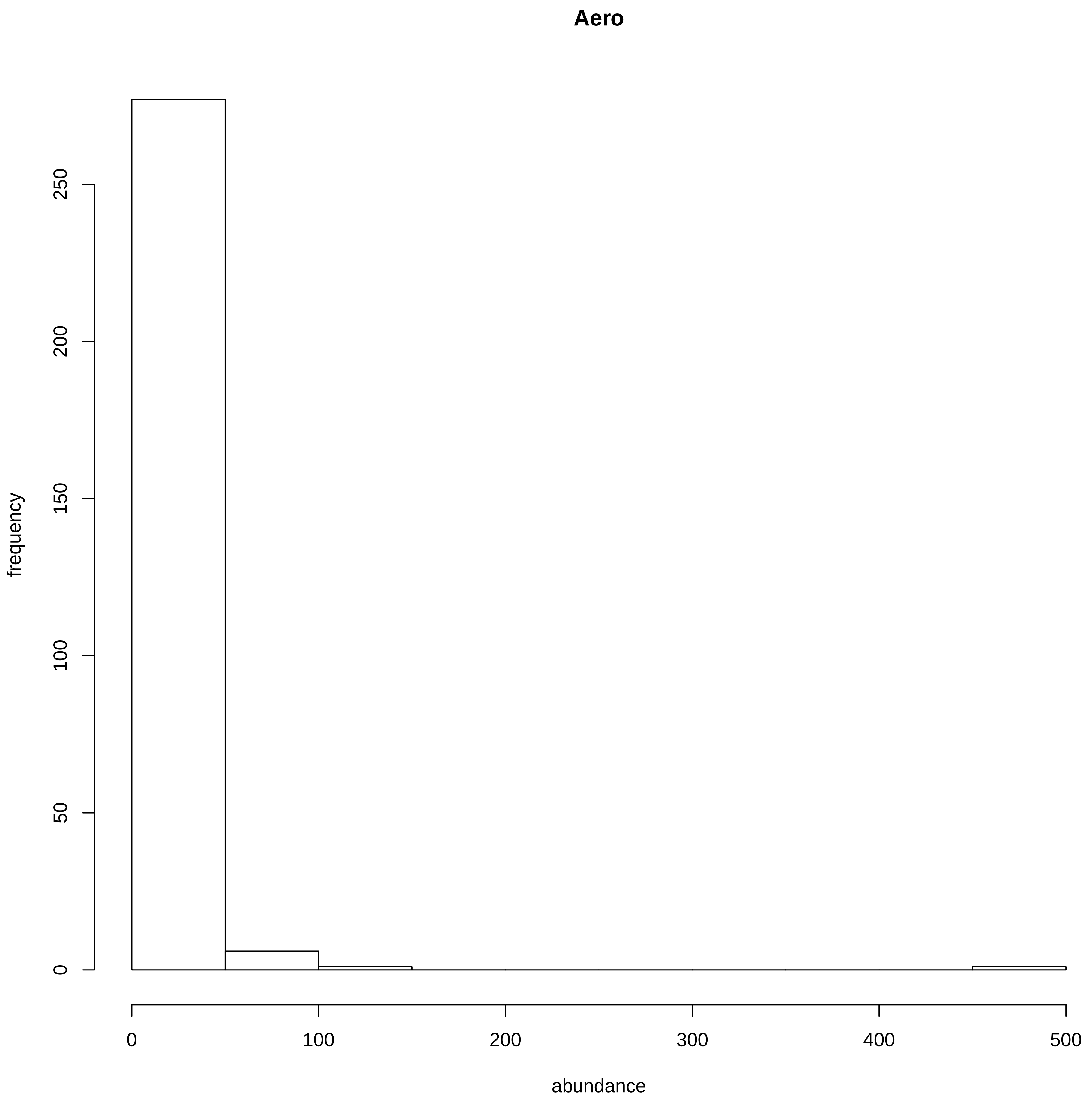
Aero
frequency
0
50
100
150
200
250
0
100
200
300
400
500
abundance

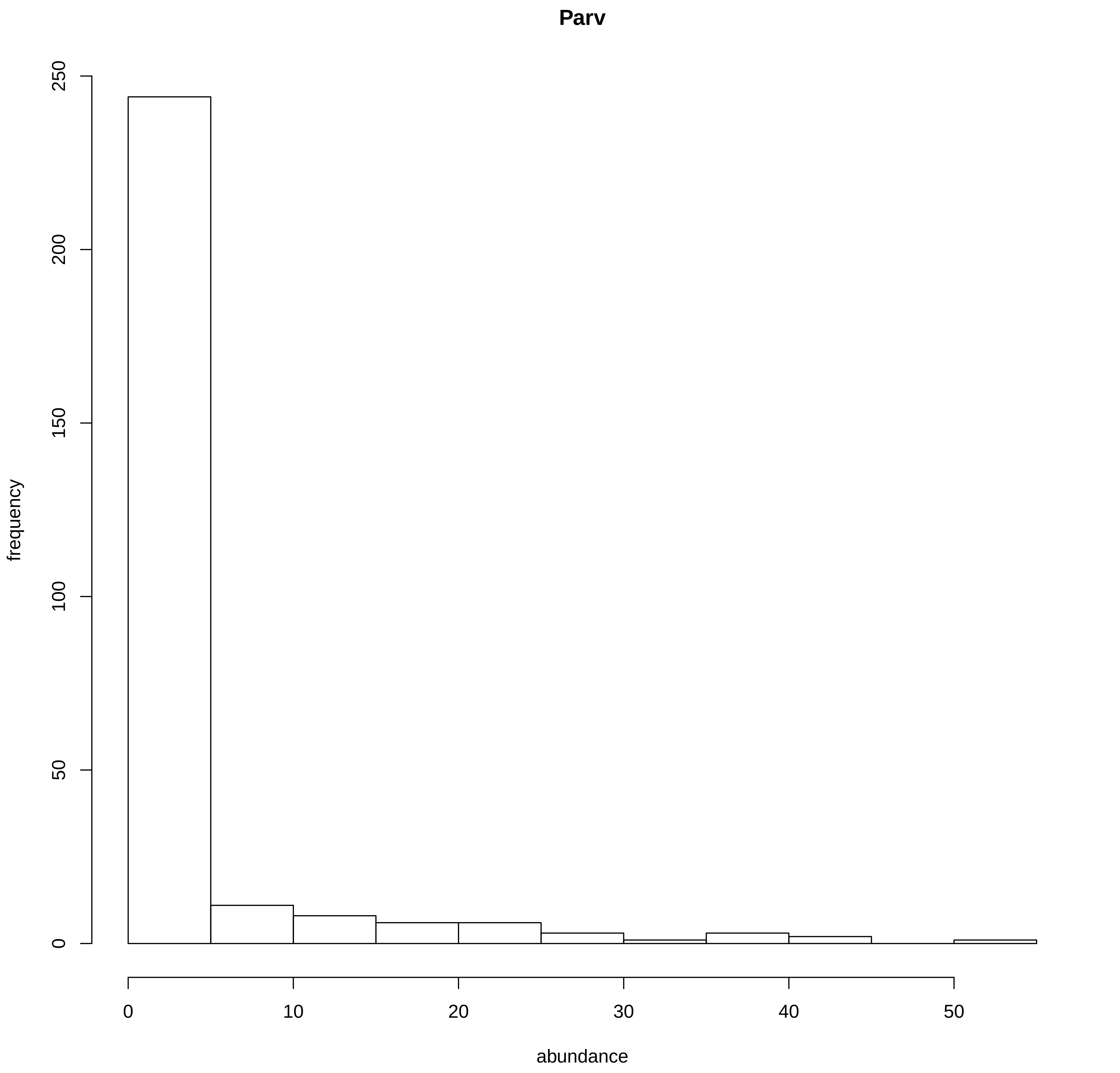
Parv
frequency
0
50
100
150
200
250
0
10
20
30
40
50
abundance

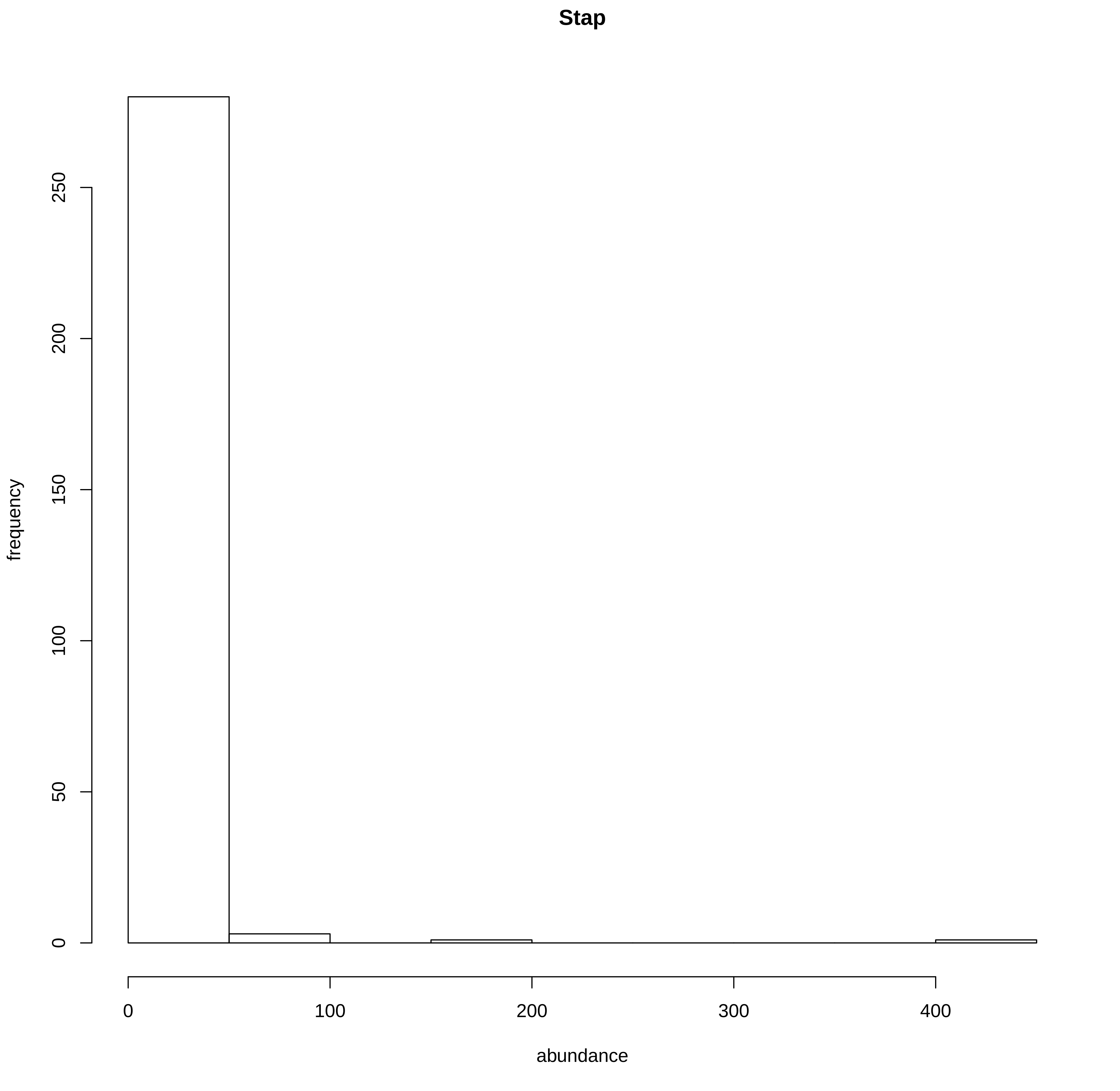
Stap
frequency
0
50
100
150
200
250
abundance
0
100
200
300
400

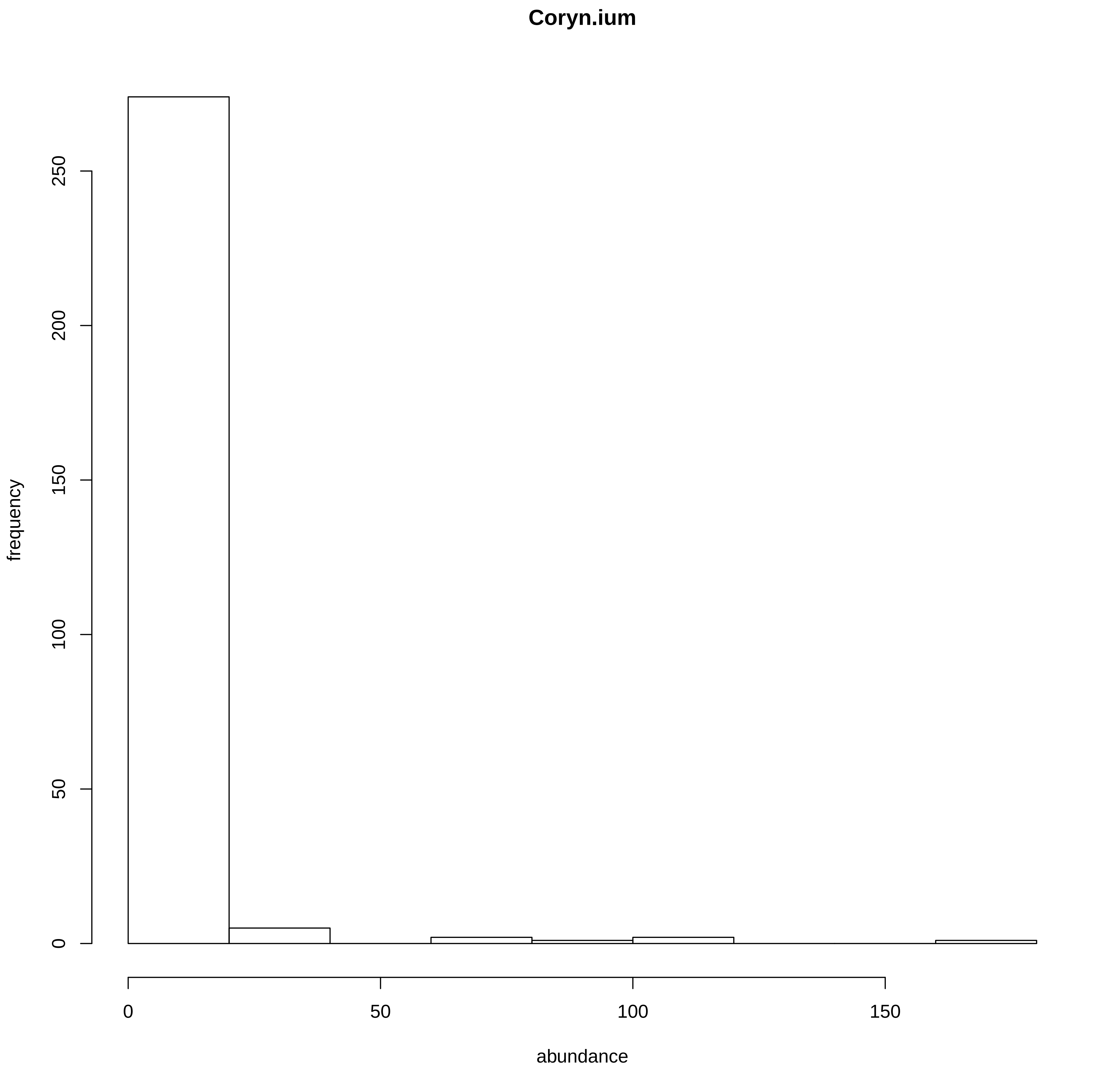
Coryn.ium
frequency
0
50
100
150
200
250
0
50
100
150
abundance

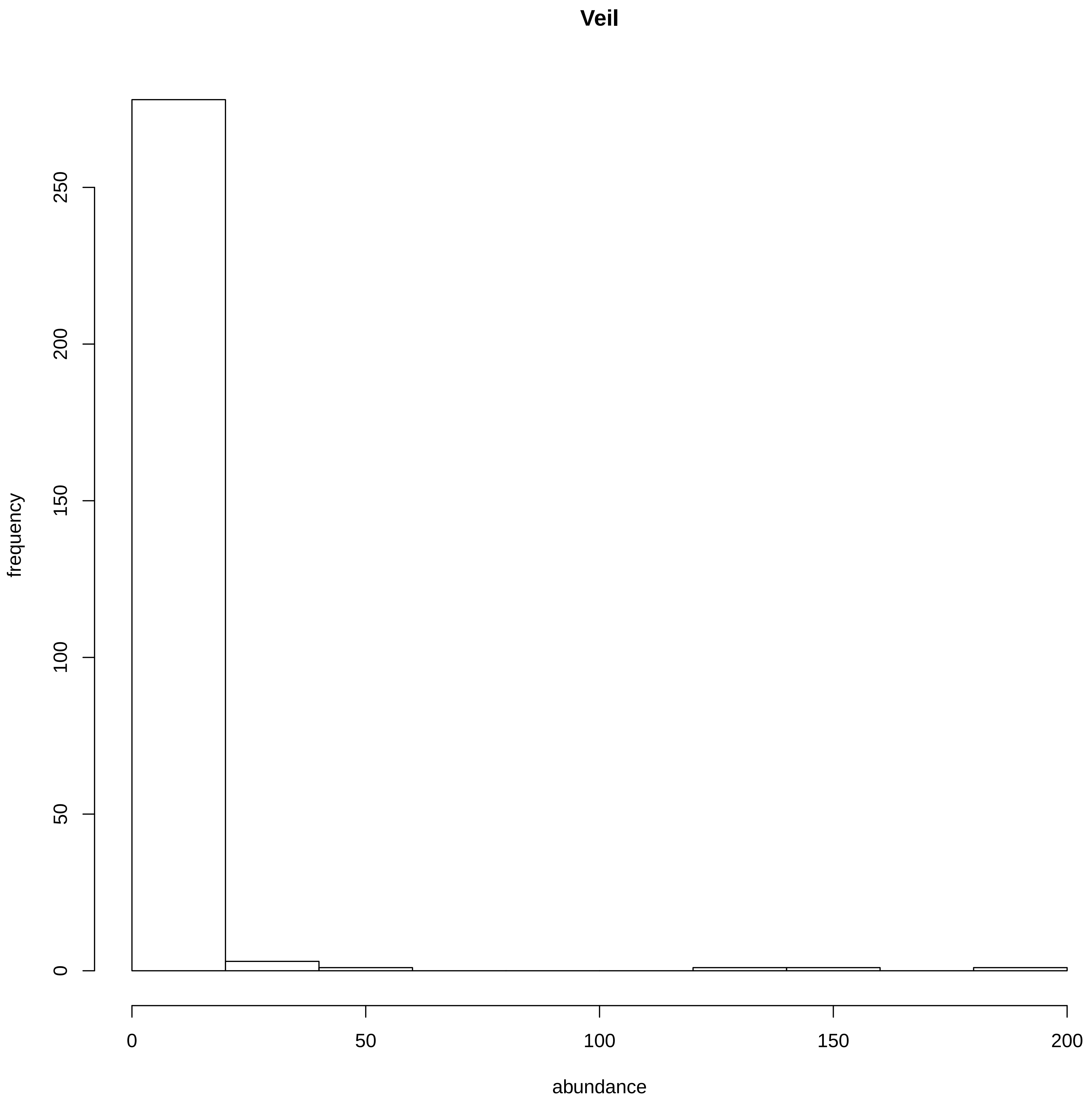
Veil
frequency
0
50
100
150
200
250
abundance
0
50
100
150
200

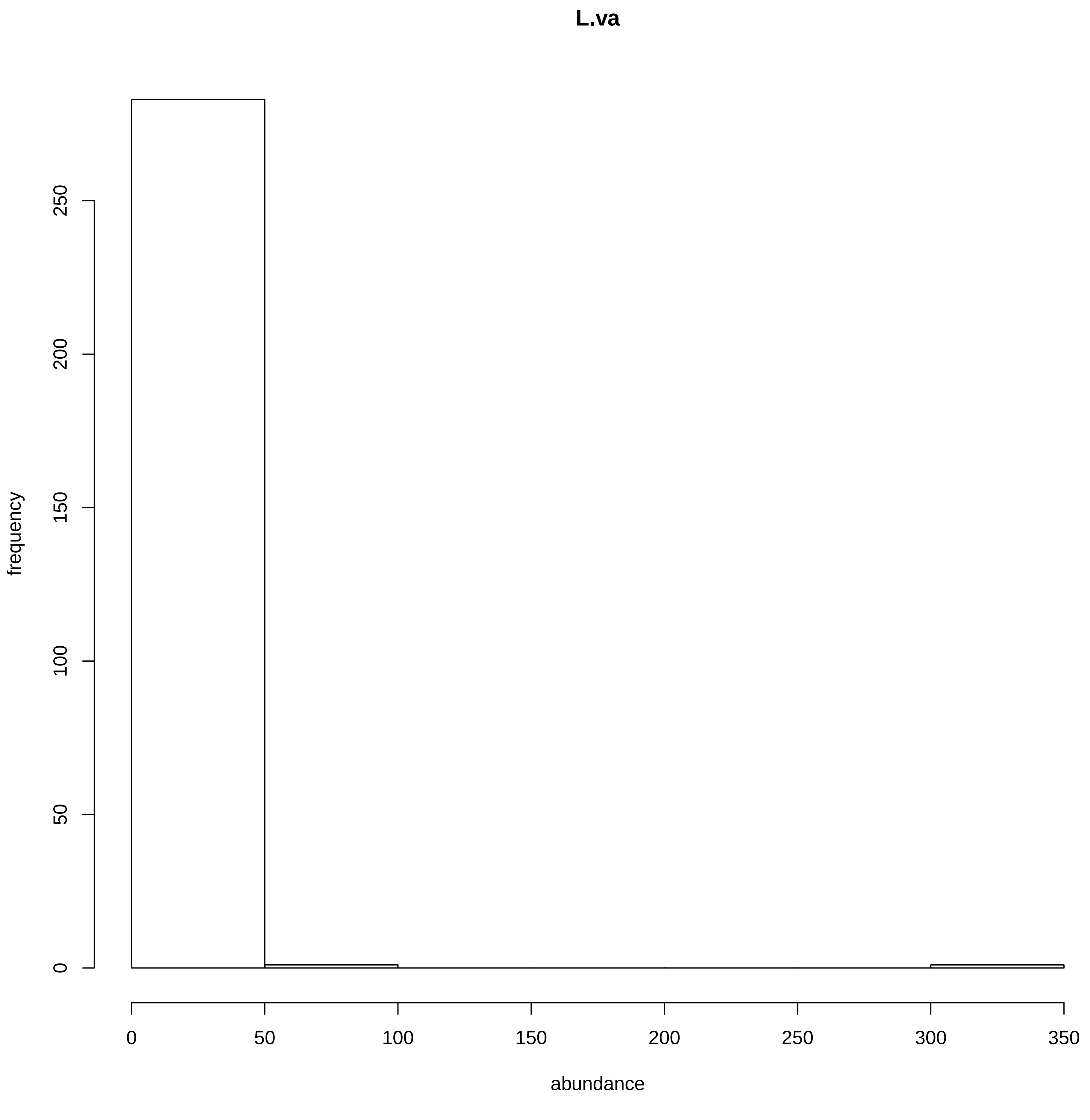

L.va
frequency
0
50
100
150
200
250
0
50
100
150
200
250
300
350
abundance

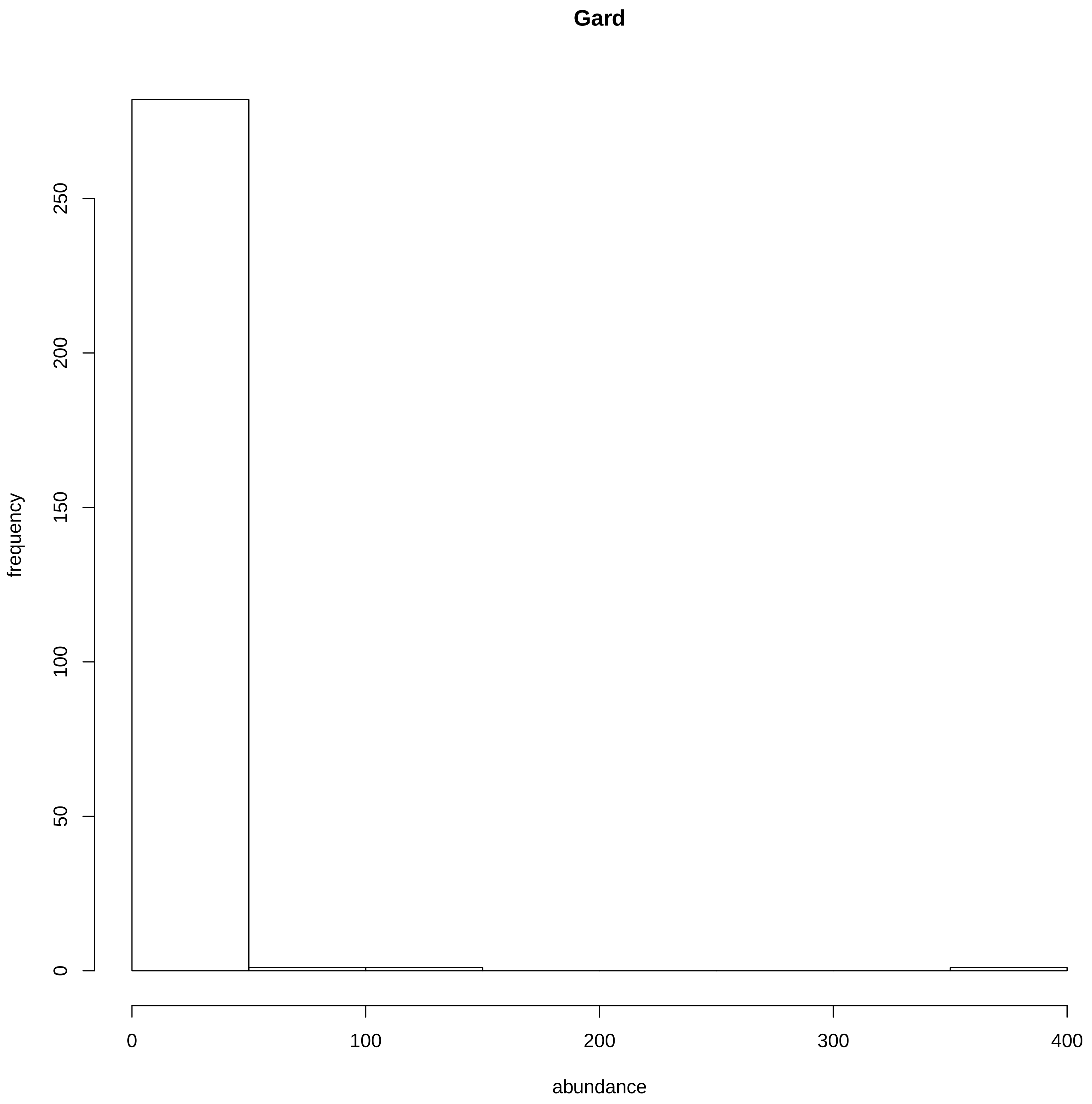

Gard
frequency
0
50
100
150
200
250
0
100
200
300
400
abundance

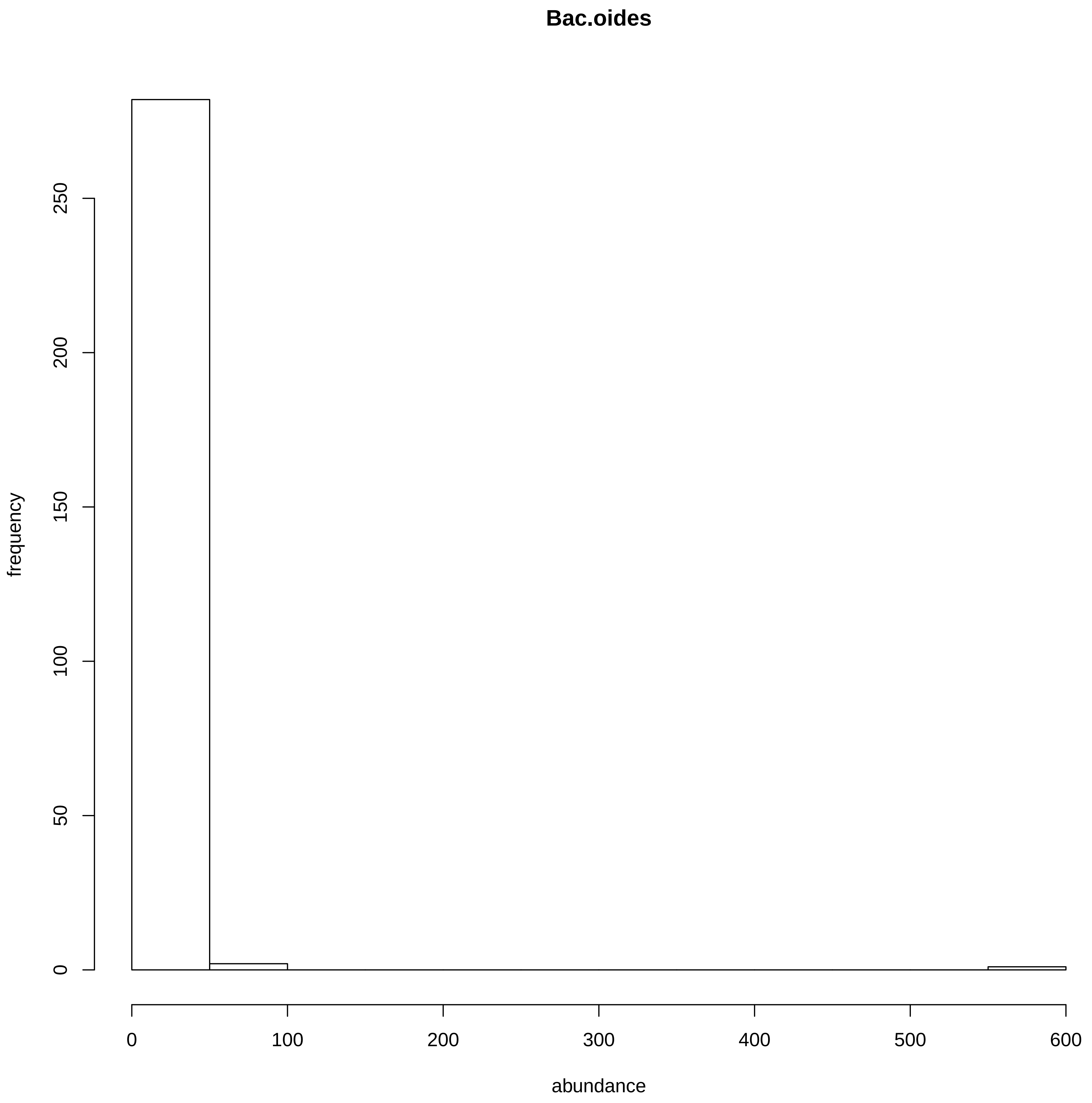
Bac.oides
frequency
0
50
100
150
200
250
0
100
200
300
400
500
600
abundance
